# Co-inoculation of *Trichoderma viride* with *Azospirillum brasilense* could suppress the development of *Harpophora maydis*-infected maize in Egypt

**DOI:** 10.3389/fpls.2024.1486607

**Published:** 2025-02-06

**Authors:** Rasha M. Elmeihy, Omar A. Hewedy, Maryam S. Alhumaidi, Khadijah A. Altammar, Eman O. Hassan, Samah A. El-Debaiky

**Affiliations:** ^1^ Department of Agricultural Microbiology, Faculty of Agriculture, Benha University, Benha, Egypt; ^2^ Department of Genetics, Faculty of Agriculture, Menoufia University, Menoufia, Egypt; ^3^ Department of Biology, College of Science, University of Hafr Al Batin, Hafr Al Batin, Saudi Arabia; ^4^ Department of Plant Pathology, Faculty of Agriculture, Benha University, Benha, Egypt; ^5^ Botany Department, Faculty of Science, Tanta University, Tanta, Egypt

**Keywords:** *Trichoderma viride*, *Azospirillum brasilense*, *Harpophora maydis*, secondary metabolites, maize, late wilt

## Abstract

Plant diseases caused by fungal pathogens are responsible for severe damage to strategic crops worldwide. Late wilt disease (LWD) is a vascular disease that occurs late in maize development. *Harpophora maydis*, the causative agent of maize LWD, is responsible for significant economic losses in Egypt. Therefore, the aim of this study was to control LWD of maize using an alternative approach to reduce the use of chemical pesticides. A combination of *Trichoderma viride*, a fungal biocontrol agent, and *Azospirillum brasilense*, a bacterial endophytic plant growth promoter, was applied *in vitro* and *in planta*. *T. viride* showed high mycoparasitic potential against *H. maydis via* various antagonistic activities, including the production of lytic enzymes, secondary metabolites, volatile compounds, and siderophores. *A. brasilense* and *T. viride* filtrates were also shown to suppress *H. maydis* growth, in addition to their ability to produce gibberellic and indole acetic acids. A significant change in the metabolites secreted by *T. viride* was observed using GC/MS in the presence of *H. maydis*. A field experiment was conducted on susceptible and resistant hybrids of maize to evaluate the antagonistic activity of *T. viride* combined with *A*. *brasilense* on LWD incidence as well as plant growth promotion under field conditions. The data revealed a significant decrease in both disease incidence and severity in maize plants treated with *T. viride* and/or *A. brasilense*. Further, there was a noticeable increase in all plant growth and yield parameters. An anatomical examination of the control and inoculated maize roots was also reflective of plant responses under biotic stress. Taken together, the obtained results provide successful eco-friendly management strategies against LWD in maize.

## 1 Introduction

The future development of sustainable agriculture is one of the backbones of the national economy in Egypt. In 2015, the Egyptian Government initiated a national project to reclaim 1.5 million acres to increase agricultural production of the strategic crops (i.e., wheat (*Triticum aestivum* L.), rice (*Oryza sativa*), and maize (corn, *Zea mays* L.)) (Moghazy and Kaluarachchi, 2020). Maize is one of the most stable foods and cereal crops in the world and is the third leading cereal crop after rice and wheat in cultivated area and productivity (Chinaru Nwosu et al., 2015; He et al., 2024). In addition, maize is an economically important crop in Africa, which is severely affected by many fungal pathogens (Veenstra et al., 2019; Benjamin et al., 2024). Late wilt disease (LWD) or black bundle disease (the causal agent of the maize LWD) is a severe vascular disease of maize caused by *Harpophora maydis* fungus, which is implicated in the PFSR complex. *H. maydis* is a soil- and seed-borne fungus related to the root-infecting species (Samra et al., 1962; Gams, 2000; Singh et al., 2020). The general symptoms include rapid and visible wilting of maize plants before tasseling, which continues until maturity. Moreover, the leaves between the veins change to a pale green before the whole leaf rolls. This disease phenotype gradually progresses from lower to upper leaves. Some plants develop yellow, purple, or dark brown streaks that appear on the lower stem, which then dry up and become shrunken. Subsequently, vascular bundles in the stalk turn reddish-brown, and internodes become discolored (Drori et al., 2013; Degani and Dor, 2021; Degani, 2022). This disease has been designated as “late wilt” because of the delayed appearance of initial symptoms until flowering, with no cobs in severe cases and undeveloped seeds (Samra et al., 1962; Payak et al., 1970; Gams, 2000; Molinero-Ruiz et al., 2010). Furthermore, the dormant sclerotia of this phytopathogenic fungus remains in the soil for many years, where it continues to colonize and infect maize roots (Drori et al., 2013). Ultimately, the fungus causes seed rot and delayed seedling emergence (Payak et al., 1970). The disease is considered the most severe threat to commercial maize production in Egypt (El-Naggarr et al., 2015). The first case of LWD disease was identified and reported in Egypt in 1961–1962, which affected 70% of the susceptible varieties (Samra et al., 1963; Johal et al., 2004) and gradually reported in other maize-growing countries such as Portugal and Spain (Ortiz-Bustos et al., 2015). *H. maydis* has recently become a significant problem in Egypt due to transmission by seeds and survival as sclerotia on corn debris. Importantly, infected seeds, crop residues, high temperature, and low humidity are the main factors affecting the distribution and development of LWD in maize. Numerous attempts have been made to reduce LWD development with integrated disease management strategies. These include the introduction of new agricultural practices, biological control strategies, physical interventions (e.g., solar heating), and chemical fungicides to protect susceptible maize varieties (Tej et al., 2018; Degani et al., 2018; Degani and Dor, 2021). However, excessive chemical fungicides (e.g., Azoxystrobin) negatively impact global health and sustainable food production. Consequently, biological control strategies have gained increased importance as an alternative environmentally friendly approach for LWD control. Interestingly, diverse beneficial microbes (i.e., *Bacillus subtilis*, *Pseudomonas koreensis*, and *Trichoderma* species) were applied as an alternative method to control LWD (Elshahawy and El-Sayed, 2018; Ghazy and El-Nahrawy, 2021). *Trichoderma* (Hypocreales) fungus is widely regarded as the most common fungal biocontrol agent for plant health management, including for ubiquitous species localized in diverse habitats (Nakkeeran et al., 2021; Woo et al., 2023). *Trichoderma viride*, *T. harzianum, T. atroviride*, *T. virens*, *T. hamatum*, and *T. longibrachiatum* have been developed as promising biological control agents due to their significant antagonistic potential (Jiang et al., 2011; Błaszczyk et al., 2016; Ghasemi et al., 2020; Cai and Druzhinina, 2021; (Dutta et al., 2023; Contreras-Cornejo et al., 2024; Guzmán-Guzmán et al., 2024; Santoyo et al., 2024))*. H. maydis*, like most fungi controlled by *Trichoderma* spp. have cell walls that contain chitin as a structural backbone and laminarin (ß-1, 3-glucan) as a filling material (Ulhoa and Peberdy, 1991). *Trichoderma* can penetrate fungal cell walls and grow extensively within mycelium by destroying their cell walls. This mechanism of action shows that the fungus produces chitinase and ß-1,3 glucanase enzymes (Chen et al., 2016; Ghasemi et al., 2019). *T. viride* can antagonistically affect plant-pathogenic fungi and nematodes, as well as improve crop resistance and promote plant growth *via* bioactive substances (Druzhinina et al., 2018; Kubicek et al., 2019; Abdelaziz et al., 2023). *Trichoderma* can produce hundreds of antimicrobial secondary metabolites, including trichomycin, gelatinomycin, chlorotrichomycin, and antibacterial peptides (Maruyama et al., 2020; Tamizi et al., 2022). These secondary metabolites can act as antibacterial agents and promote plant growth (Nawrocka et al., 2023). Importantly, there is a lack of data regarding the antagonistic activity of *Trichoderma* against LWD. The antifungal and anti-mycotic activities of *Trichoderma viride* and *Trichoderma harzianum* against different pathogenic fungal strains have previously been evaluated *in vitro* using a dual culture assay (Yogalakshmi et al., 2021; Hossain and Sultana, 2024). *T. viride* was shown to have an effective, potent activity for suppressing the mycelial growth of diverse pathogens, including *Curvularia lunata*, *Exserohilum rostratum*, *Fusarium chlamydosporum*, *Fusarium incarnatum*, *Fusarium proliferatum*, and *Macrophomina phaseolina* (Yassin et al., 2021). Moreover, eight *Trichoderma* isolates were tested as biocontrol agents against *M. maydis*. *T. longibrachiatum* and *T. asperelloides* showed high mycoparasitic activity against the pathogen by producing soluble metabolites that inhibit or kill the maize pathogen (Degani and Dor, 2021). *Azospirillum* is a type of *Rhizobacteria* and an associative nitrogen fixer (diazotroph) that comprises seven species, i.e., *A. amazonense*, *A. brasilense*, *A. doebereinnerae*, *A. halopraeferens*, *A. irakense*, *A. largimobile*, and *A. lipoferum*. *A. brasilense* is an aerobic bacterium that exhibits the main characteristics that define plant growth-promoting, such as nitrogen fixation and siderophore production (Galindo et al., 2020). It has been reported that *Azospirillum* strains have the capability to produce different phytohormones, including indole acetic acid (IAA), cytokinins, gibberellins, and other compounds, such as polyamines and amino acids. Notably, the inoculation of *Azospirillum brasilense* represents a potentially efficient method to improve plant development (Mehnaz, 2014; Fukami et al., 2018; Cassán et al., 2020; Rabani et al., 2023; Gureeva and Gureev, 2023). The attachment of *Azospirillum* to the roots is considered the first necessary step for the colonization of the host plants, which mainly colonizes the root surface (Steenhoudt and Vanderleyden, 2000; Pedraza et al., 2020; Yadav et al., 2024). *Azospirillum* species are able to colonize hundreds of plant species and improve their growth, development, and productivity, such as maize (Fukami et al., 2016; Cardozo et al., 2022). Interestingly, *Azospirillum brasilense* Sp7 and a bio-control fungus (*Trichoderma harzianum* Rifai 1295-22), were evaluated for their single and combined effects on dry bean (*Phaseolus vulgaris*) and wheat (*Triticum aestivum* L.) grown in soil (Öğüt et al., 2005). A field experiment was carried out to evaluate the feasibility of inoculating rice seedlings with biofertilizers (*Azospirillum* and *Trichoderma*) to reduce the use of chemical inorganic nitrogen (N) fertilizer on rice (Khan, 2018). Hence, this study aimed to use *Trichoderma viride* as a biocontrol agent to control LWD of maize plants caused by *H. maydis*-infected maize in the presence of *Azospirillum*, as a plant-growth promoter already known for its ability to associate with cereal crops such as maize.

## 2 Materials and methods

This study was divided into two parts; the first was conducted *in vitro*, while the second utilized microbial application in the field (Summer, 2020).

### 2.1 Biological materials

**Figure 1:**
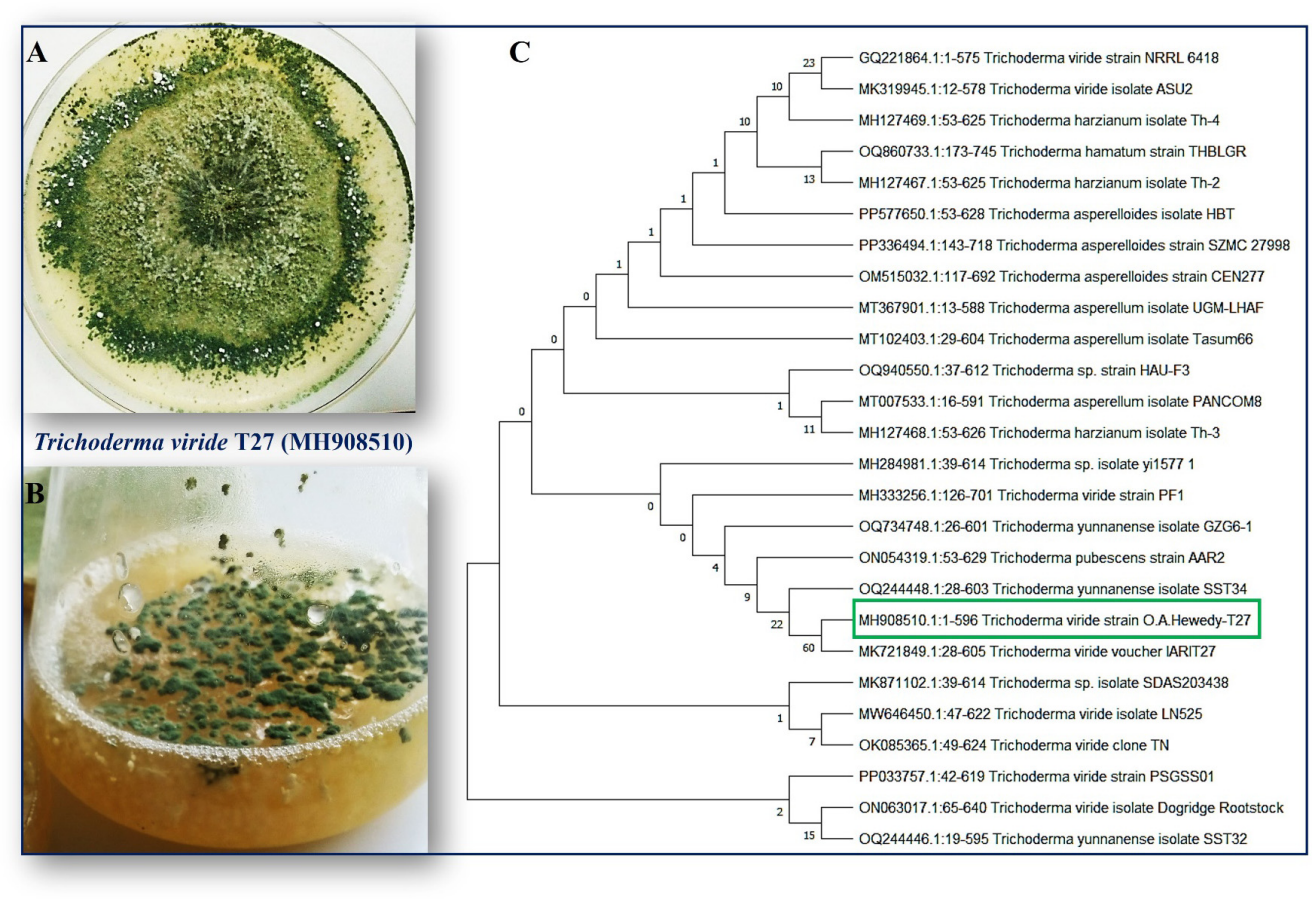
**(A)** Colonies of endophytic fungi *T. viride* grown on potato dextrose agar (PDA) media under photoperiod conditions at 28°C for five days show a ring around the original inoculum. **(B)** *T. viride* grown on liquid media potato dextrose broth (PDB) under photoperiod conditions at 150 rpm. **(C)** A phylogenetic tree was inferred through a maximum-likelihood analysis of aligned rDNA internal transcribed spacers ITS (ITS4 and ITS5) sequences from different *Trichoderma* isolates using MEGA11.0. *T. viride* T27 (MH908510) was identified based on the maximum likelihood model and their closest matches, followed by the GenBank accession number. The numbers above the branches indicate the number of times the group consisting of the species to the right of that fork occurred among the tree out of 100 trees. The boxed species indicate the phylogenetic position of *Trichoderma viride* (T27) compared with other fungal strains deposited on the GenBank.

*T. viride* strain T27 (accession number MH908510) was isolated and identified in a previous study (Hewedy et al., 2020b), as presented in (**Figures 1A–C**). *H. maydis* (isolate C5) was kindly supplied by the Department of Plant Pathology, Faculty of Agriculture, Benha University, Egypt. All fungi were propagated on potato dextrose agar (PDA; HIMEDIA Co.) at 28°C for five days and then maintained at 4°C until further testing.

*A. brasilense* (strain MC12) was obtained from the Department of Agricultural Microbiology, Faculty of Agriculture, Benha University, Egypt, and cultured on nutrient broth medium (HIMEDIA Co. M002, 13.0 g in 1000 mL ddH_2_O) at 28°C ± 2°C for four days before storage at 4°C until subsequent testing.

### 2.2 *In vitro* studies

#### 2.2.1 Dual culture

The antagonistic activity of *Trichoderma* (T27, MH908510) was evaluated *in vitro* against *H. maydis* using the dual culture technique at 28°C ± 2°C (Pan and Bhagat, 2008). The pathogen inhibition percentage (IP) against mycelial growth was calculated according to the following formula (Hajieghrari et al., 2008):


$$\text{IP }(\%)=\frac{\text{C}-\text{T}}{\text{C }}\text{ x }100$$

where, C = radial growth in control (pathogen only), T = radial growth in treatment.

Next, the hyphal interaction in the contact area was examined and photographed using a light microscope equipped with a USB camera at a magnification power of 800× (OPTIKA C-B5 5.1 Megapixel CMOS USB 2.0 Camera, Microscope Company, USA).

#### 2.2.2 Bioagent filtrate activity against *H. maydis*

The antifungal activity of the culture filtrate of T27 against *H. maydis* was tested *in vitro* as described by (Elshahawy and El-Sayed, 2018). *Trichoderma* T27 was grown for ten days at 28°C ± 2°C with agitation (150 rpm) in 100 mL Erlenmeyer flasks containing 50 mL of sterilized PDB. Next, the mycelial growth was removed by filtration using filter paper (Whatman filter paper #1, WHA1001090) and centrifuged at 5000 rpm with slight modifications. Subsequently, 5 mL of *Trichoderma* filtrate was added to 45 mL of PDB medium to make a final concentration of filtrate (10% v/v). This was performed in triplicate, and a negative control was prepared using 5 mL sterile distilled water instead of fungal filtrate (Rahman et al., 2023).

The amended flasks were then inoculated with a five-day-old (5 mm) disk of *H. maydis* and incubated at 28°C with shaking at 150 rpm for nine days. All flasks were weighed at zero time and after 3, 6, and 9 days of inoculation. The reduction of *H. maydis* growth was measured according to the following equation:


$$\text{Reduction }(\%)=\frac{\text{Wc}-\text{Wt}}{\text{Wc}}\text{x }100$$

where, *Wc* = weight of control flask, *Wt* = weight of treatment flask at the same time as control.

The synergistic effect of culture filtrates of T27 and *A. brasilense* was also studied. Briefly, 5 mL of bacterial culture (*A. brasilense*) was transferred to 100 mL Erlenmeyer flasks containing 50 mL of Dobereiner’s broth (DB) medium and incubated at 28°C ± 2°C for four days. The microbial cells were then removed from each culture by filtration through filter paper (Whatman no. 1) and then centrifugation (centrifuge Tube GKF, China) at 5,000 rpm for 10 min to obtain cell-free filtrate for further experiments (Abdulkareem et al., 2014).

Subsequently, 5 mL of bacterial filtrate was mixed separately with 45 mL of PDB. In comparison, 2.5 mL of bacterial filtrate, 2.5 mL of the T27 filtrate, and 45 mL of PDB were prepared for a final concentration of 10% (v/v). Sterilized DB medium and distilled water served as the negative control. Next, control and treatment flasks were inoculated separately with 5 mm diameter mycelial discs of *H. maydis* and incubated at 28°C ± 2°C with shaking at 150 rpm. The weight of each flask was measured at zero time and after 3, 6, and 9 days of inoculation. The reduction in *H. maydis* growth was calculated as described in the equation above. All treatments and controls were performed in triplicate.

#### 2.2.3 Gas chromatography/mass spectrometry analysis of secondary metabolites

Mono- and dual cultures of *T. viride* T27 alone and *Trichoderma* in the presence of *H. maydis* C5 were tested to produce bioactive secondary metabolites. *T. viride* (T27) was grown separately or in combination on a PDB medium at optimum temperature for 20 days. Cultures were then filtered through filter papers (Whatman no. 1.), and equal volumes of filtrate and hexane (1:1 v/v) were mixed gently to extract the metabolites. Subsequently, hexane was evaporated using a rotary evaporator with a rotor speed of 120 rpm at 400 °C until the precipitate was formed. The precipitate was re-suspended in acetone for further characterization by GC/MS (GCMS-QP2010 Plus ultra), as previously described by (Bhardwaj and Kumar, 2017).

#### 2.2.4 Volatile compounds and siderophores

Ammonia (NH_3_) and hydrogen cyanide (HCN) produced from T27 culture filtrate were estimated spectroscopically according to previously described methods. Briefly, a fresh culture of T27 was prepared in a test tube containing 10 mL of peptone water and incubated for five days at 28°C ± 2°C. After incubation, 1 mL of the culture was transferred to Eppendorf and combined with 50 μL of Nessler’s reagent, which was prepared by mixing 2 g KI in 5 mL of H_2_O. Next, 3 g of HgI_2_ was added, and the resulting solution was made up to 20 mL. Finally, 40 g of KOH (30%) was added to provide the alkaline base. A color change from a clear solution to a faint yellow indicated the presence of a small amount of ammonia, while a deep yellow or brown color was indicative of higher ammonia content. The color change was measured using a spectrophotometer (Sco. Tech, SP UV-19) at 450 nm. A standardized curve was generated by titrating ammonium sulfate from 0.1 – 5 μmol/mL (Cappuccino and Sherman, 1992; Reetha et al., 2014; Abdenaceur et al., 2022).

Next, 10 mL of T27 was inoculated in a 100 mL Erlenmeyer flask containing King’s B broth medium amended with 4.4 g/L glycine to detect HCN as previously described (El-Rahman et al., 2019). Non-inoculated flasks were used as negative controls, and all treatments and controls were performed in triplicate. Sterilized filter paper strips dipped in picrate solution (0.5% picric acid in 2% sodium carbonate) were attached to the neck of the flasks. Each flask was then plugged and sealed off with Parafilm and incubated with shaking at 140 rpm for four days at 28 °C ± 2°C. A change in the color of the filter paper strips from yellow to light brown, brown, or brick red was recorded as a weak (+), moderate (++), or strong (+++) reaction, respectively. A lack of color change was recorded as a negative (−) reaction. Moreover, the color intensity was detected and measured by spectrophotometry at 625 nm by dipping the filter paper strips into 10 mL of distilled water to elute the produced color. Likewise, qualitative and quantitative assessment of siderophores using chrome azurol S (CAS) reagent was also performed (Payne, 1994; Dutta et al., 2015).

For the qualitative evaluation, T27 was grown on CAS agar plates at 28°C for five days. Siderophore production was detected when the color of the medium changed to orange color. The CAS-shuttle assay was used for quantitative detection of siderophores. A total of 0.5 mL of supernatant was obtained from the filtration of broth cultures of the tested T27, which was mixed with an equal volume of CAS reagent. Siderophores were analyzed by spectrophotometry at 630 nm, where uninoculated broth medium was used as control, and the proportion of siderophore units was calculated as a percentage according to the following formula:


$$\text{\% siderophore units}=\frac{\text{Ac }-\text{ As}}{\text{Ac }}\text{ X }100$$

where, *Ac* = absorbance of the control, *As* = absorbance of the sample.

#### 2.2.5 Lytic enzymes of T27

Chitinase (3.2.1.14) activity was qualitatively estimated using the Lukewarm agar medium, amended with bromocresol purple for colored zone formation. The constituents of the medium were as follows: MgSO_4_.7H_2_O, 0.3 g/L; (NH_4_)_2_SO_4_, 3; KH_2_PO_4_, 2 g/L; citric acid monohydrate,1 g/L; agar, 15 g/L; colloidal chitin, 4.5 g/L; bromocresol purple, 0.15 g/L; and 200 μL Tween-80, pH 4.7 (Sigma-Aldrich, USA). Solidified medium plates were inoculated with T27, incubated at 28°C ± 2°C for seven days, and observed for colored zone formation (Agrawal and Kotasthane, 2012). Moreover, for quantitative assessment of chitinase, T27 was inoculated in Lukewarm broth medium without bromocresol purple and incubated in a shaker at 28°C with shaking at 150 rpm for five days. After the incubation period, the culture was centrifuged at 5000 rpm for 15 min, and chitinolytic activity was quantitatively assayed in culture filtrate by measuring the released reducing sugars from colloidal chitin. Briefly, 0.3 mL of 1 M sodium acetate buffer (pH 4.6) and 0.2 mL of colloidal chitin were transferred to a test tube containing 1 mL of culture filtrate, then incubated at 40°C for 20 h. After incubation, the mixture was centrifuged at 10,000 rpm for 5 min. Next, 0.75 mL of the tested mixture was combined with 0.25 mL of DNS solution (i.e., 1.0 g of 3,5 dinitro salicylic acid in 20 mL 2 M NaOH, to which 30 g of sodium potassium tartrate was slowly added before dilution to a final volume of 100 mL using distilled water) were mixed in test tubes and heated at 100°C for 5 min. After cooling, the absorbance was detected at 582 nm using a spectrophotometer (Sco. Tech, SP UV-19) (Miller, 1959). A standard N-acetyl-glucosamine (NAGA) curve was used to calculate chitinolytic activity using the concentration of released NAGA as the readout. Both amylase and cellulase activities were estimated in crude culture filtrate of T27. Starch broth and carboxy methyl cellulose media were used to estimate amylase and cellulase. The dinitrosalicylic acid method was applied for both enzymes to measure the released amounts of glucose by spectrophotometry at 575 nm using a glucose standard curve as previously described (Miller, 1959).

#### 2.2.6 Growth hormones of *T. viride* (T27) and *A. brasilense*

Indeed, most endophytic microbes, either fungi or bacteria, live in association with the roots of many plants. The ability of *Trichoderma* T27 and *A. brasilense* to produce indole acetic acid (IAA) and gibberellic acid (GA_3_) *in vitro* was assessed using Salkowski’s and Folin–Ciocalteu (FC) reagents as previously described (Crozier et al., 1988; Pastrana et al., 1995; Perrig et al., 2007; Spaepen et al., 2008; Zhang et al., 2013; Nieto-Jacobo et al., 2017; Abdenaceur et al., 2022). T27 and *A. brasilense* cultures were grown for seven days on Czapek-dox broth and nutrient broth media supplemented with L-tryptophan (1 mg/L), respectively. After the incubation period, the microbial growth was removed by filtration. Next, 20 mL of each culture filtrate was centrifuged at 3000 rpm for 5 minutes. IAA production was then tested by adding 2 mL of the filtrate to 2 mL of Salkowski reagent (0.5 M ferric chloride (FeCl_3_) and 35% perchloric acid (HClO_4_)) and allowed to stand for 15 minutes. A color change to pink (measured at 535 nm) was indicative of a positive result. Additionally, for the GA_3_ assay, 1 mL of microbial supernatant was combined and boiled with 1 mL of the reagent, 1 mL of concentrated HCl, and 3 mL dH_2_O for 5 min in a water bath. Finally, after cooling to room temperature, the produced color change from green to blue was measured at 750 nm using a spectrophotometer (Sco. Tech, SP UV-19).

#### 2.2.7 Mycoparasitism activity assay

Mycoparasitism activity was studied as previously described (Bhat, 2017; Mukherjee et al., 2022) with minor modifications. Briefly, 15 mL of PDA was poured into a 90 mm petri dish and allowed to solidify. Then, 5 mm discs of *T. viride* (T27) and *H. maydis* C5 were inoculated at opposite points on the edges of PDA agar and then incubated for 5–7 days at 28°C, allowing the two fungi to grow toward each other. Upon interaction, a small portion was carefully separated without destroying the interacting mycelia and transferred to a clean glass slide. The interfering mycelia were then examined under a light microscope (800×) with a USB camera (OPTIKA C-B5 5.1 Megapixel CMOS USB 2.0 Camera, Microscope Company, USA).

### 2.3 Field experiments

#### 2.3.1 Plant cultivars and field experimental design

The results of the *in vitro* studies on maize plants (*Zea mays* L.) were applied in the field during the growing season (Summer 2020). This experiment was carried out at the Faculty of Agriculture, Benha University, Egypt. The current study applied a randomized complete block design in triplicate. Each experimental plot (21 m^2^) was split into six rows. The *in planta* and field treatments were designed as follows: T1 as the uninfected control; T2 as the infected control by the pathogen *H. maydis*; T3: bioagent (*T. viride* T27 only) inoculated plants; T4: plants inoculated with a combination of (T27 + *A. brasilense*); T5: plants infected with a combination of (T27 + *H. maydis*); and T6: the last treatment which consisted of the inoculated plants with a combination of (T27 + *A. brasilense* + *H. maydis*). The grains of two yellow solitary hybrid cultivars of maize varied in their susceptibility to LWD and were purchased from Pioneer Company, Egypt (https://www.pioneer.com/landing), (**Supplementary Figure S1**). The first hybrid (11N30) was registered as a tolerant cultivar, while the second (3062) was considered susceptible. This experiment evaluated the influence of *T. viride* T27 and *A. brasilense* on the plant growth criteria and incidence of LWD caused by *H. maydis*. The soil was clay loam comprised of 1.52% organic matter (pH 8.2).

#### 2.3.2 Cultivation, pathogenicity, and antagonistic experiments

Next, we tested antagonistic microorganisms following inoculation. Briefly, *T. viride* (27) was prepared on PDB medium at 28 °C ± 2°C with shaking (150 rpm) for seven days before it was homogenized and mixed with soil one week before cultivation (excluding the treatment of the control rows). Then, *T. virdie* homogenized culture containing 10^6^ Spore/ml was added at the rate of 100 ml/Jura (10 cm and depth 10 cm). Extra doses of *T. viride* suspension were added to the plants three times during the growing season at a rate of 50 mL/plant. Further, cell suspensions of *A. brasilense* MC12 were grown on DB medium and incubated at 28°C ± 2°C for four days. Then, maize seeds were soaked for 30 min in a mixture of the cell suspension (10^8^ colony forming unit (cfu) mL^−1^) and 10% Arabic gum as an adhesive agent before cultivation. An excessive dose of *A. brasilense* inoculum was added to the soil rhizosphere near each plant three times during the growing season at a rate of 50 mL/plant.

#### 2.3.3 Cultivation process

Maize seeds were sown at a 20 cm distance between plants. After 21 days of emergence, plants were manually thinned to one plant/Jura. Plants were irrigated and chemically fertilized with a nitrogen (N; 140 kg N_2_ as ammonium sulfate), phosphate (P; 200 kg P_2_O_5_ as calcium superphosphate), and potassium (K; 50 kg K_2_O as potassium sulfate) mix as recommended by the Ministry of Agriculture and Land Reclamation of Egypt in two equal doses at vegetative and flowering stages. The dose of N was reduced to half in treatments with *A. brasilense*.

#### 2.3.4 Soil and plant infection with pathogenic fungi

*H. maydis* was cultured on PDB at 28 °C ± 2°C with shaking (150 rpm) for seven days, then used to infect maize plants as previously described (Shekhar and Kumar, 2012) with some modifications. A sterilized thin syringe was filled with homogenized *H. maydis* inoculum under sterilized conditions. Subsequently, the outer surface of the plants (40 days old) was sterilized with 70% (v/v) ethyl alcohol, and the pathogen was injected into the second lower internodes above the soil level. In addition, the infected plants were apparently observed with late wilt symptoms at 20–25 days after infection. Each experimental plot (21 m^2^) was split into six rows; Maize seeds were sown at a 20 cm distance between plants. After 21 days of emergence, plants were manually thinned to one plant/Jura.

#### 2.3.5 Disease assessment

Disease assessment was performed periodically as disease incidence and severity after 60, 80, and 120 days of sowing (or after 20, 40, and 80 days of infestation). Disease incidence and severity were periodically recorded by examining the stems and leaves of 10 randomly selected infected plants after 60, 80, and 120 days of sowing (or after 20, 40, and 80 days of infection).

Ten plants of each replicate were randomly selected and labeled with all treatments to determine disease severity. A previously published scoring scale, which is divided into six grades as follows, was used for estimating LWD severity (El-Naggarr et al., 2015):

0: No symptoms on stalk and leaves.

1: Dark green longitudinal streaks appear with healthy-appearing leaves on the first basal internode.

2: Shrinking appears on the first internode; dark green longitudinal streaks extend to the second internode, and a slight yellowing occurs on the lower leaves.

3: Shrinking extends to the second and third internodes while a few lower leaves appear slightly dry.

4: Shrinking overcomes most internodes, twists the first internode, and dries most leaves.

5: All stalk internodes and leaves are dried, and the plant has died.

#### 2.3.6 Maize growth characteristics, cone parameters, and yield

Mature maize plants were used to determine all growth parameters such as plant height (cm), number of leaves, fresh weight/plant (g), dry weight/plant (g), cone length (cm), cone diameter (cm), cone weight (g), number of rows/cones, number of grains/rows, weight of seeds/cone, weight of cones/15 plant (g) and weight of seeds/15 plant (g).

#### 2.3.7 Photosynthetic pigments

Eighty-day-old leaves were collected to extract and measure photosynthetic pigments (Nornal, 1982; Zhao et al., 2003). Different photosynthetic pigments (i.e., chlorophyll A, chlorophyll B, and carotenoids) in the leaf tissues were detected by extracting 1 g of leaf sample in 10 mL 80% (v/v) acetone for 2 mins before filtration through Whatman no. 1 filter paper, and the volume was then made up to 100 mL. Next, the optical density (OD) was measured at 660, 644, and 440 nm, and the quantity of each pigment was calculated as mg/L according to the equations:


Chlorophyll A=(9.784 x OD660)−(0.99 x OD644)

Chlorophyll B=(21.426 x OD644)−(4.650 x OD660)

Carotenoids=(4.685 x OD440)−(0.268 x (Chlorophyll A+Chlorophyll B))

#### 2.3.8 Oxidative enzymes

After 80 days of cultivation, 2.0 g of fresh maize leaves were collected and grinded using a mortar and pestle in washed and dried sand with 4 mL of 0.1 M sodium phosphate buffer (pH 7) under sterilized conditions. The samples were then filtered and centrifuged at 3000 rpm for 20 mins, and the supernatant was taken to measure oxidative enzyme activity. All measurements and assays were performed in triplicate. Polyphenol oxidase (PPO, 1.10.3.1) activity was estimated (Lippolis et al., 2008). In a clean test tube, 0.2 mL of crude enzyme extract was mixed with 1 mL of 0.2 M sodium phosphate buffer (pH 7) and 1 mL of 1 mM catechol. Then, the final volume was adjusted to 6 mL with distilled water. The reaction mixture was incubated at 30°C for 30 min, and the absorbance was detected at 495 nm (Matta and Dimond, 1963). Catalase (CAT, 1.11.1.6) activity was evaluated according to published methods (Shim et al., 2003). In a reaction mixture of 0.5 mL 0.2 M sodium phosphate buffer (pH 7.6) and 0.3 mL of 0.5% (v/v) hydrogen peroxide (H_2_O_2_), the enzyme extract (0.4 mL) was added, and the final volume was completed to 3 mL with distilled water. The decrease in H_2_O_2_ absorbance at 240 nm was monitored, and CAT activity was calculated as µmol/min/g of fresh weight. Peroxidase (PO, 1.11.1.7) activity was measured by mixing 0.3 mL enzyme extract with a reaction mixture containing 0.5 mL 0.1 M potassium phosphate buffer (pH 7), 0.3 mL 0.05 M pyrogallol, 0.1 mL 1% (v/v) H_2_O_2_ and completed to 3 mL with distilled water. H_2_SO_4_ (5% (v/v)) was used to terminate the reaction after incubation for 15 min at 25°C. Subsequently, the change in absorbance at 470 nm was monitored, and PO activity was calculated as µmol/min/g of fresh weight (Allam and Hollis, 1972). Next, phenylalanine ammonia-lyase (PAL) activity was measured by combining 3.8 mL of sodium borate buffer (pH 8.8) with 1 mL of 0.33% (w/v) L-phenyl alanine and 0.2 mL of crude enzyme. After incubation at 40°C for 15 mins, the mixture was left to cool at room temperature, and the absorbance was measured at 290 nm (Cheng and Breen, 1991). Chitinase (Chit, 3.2.1.14) activity was measured as previously described (Mauch et al., 1988) by measuring absorbance at 540 nm and reported as mM N-acetyl glucose amine released/g of fresh weight/60 mins.

#### 2.3.9 Histological assessment of maize roots

Comparative anatomical characteristics of the maize roots between treated and non-treated plants (control) were examined approximately 150 days after sowing (Johanson, 1940). Briefly, root cross-sections were taken from the primary roots and examined for histological changes. Maize primary roots were collected from each plant during the flowering stage and fixed in FAA (5 mL formalin, 5 mL glacial acetic acid, and 90 mL 70% (v/v) ethyl alcohol). Next, the samples were prepared for analysis with some modifications according to (Sass, 1951). Briefly, the samples were washed in 50% (v/v) ethyl alcohol, dehydrated in serial dilutions of ethyl alcohol (70, 90, 95, and 100% (v/v)), incubated in xylene, embedded in paraffin wax with a melting point of 60°C–63°C, sectioned to 12 μm sections, double-stained with fast green and safranin, cleared in xylene, and finally, mounted in Canada balsam. Next, root cross-sections were examined for histological changes. The prepared sections were examined, counted, and measured under a light microscope with an optical camera (magnification power is 100×).

### 2.4 Statistical analyses

All experiments were conducted using factorial and a completely randomized design with three replicates for each experiment, considering the corn variety (S&R) and the different treatments as factors A and B. A two-way analysis of variance (ANOVA) was performed for all parameters using GraphPad Prism Software version 9.3.1 (GraphPad Software, USA) and SPSS v.28. All error bars shown represent the range of data points. Means were compared using Duncan multiple range test DMRT at a 95% significance level (p ≤ 0.05) (Duncan, 1955; Gomez, 1984).

## 3 Results

### 3.1 *In vitro* experiments

#### 3.1.1 Dual culture and microscopy data

**Figure 2:**
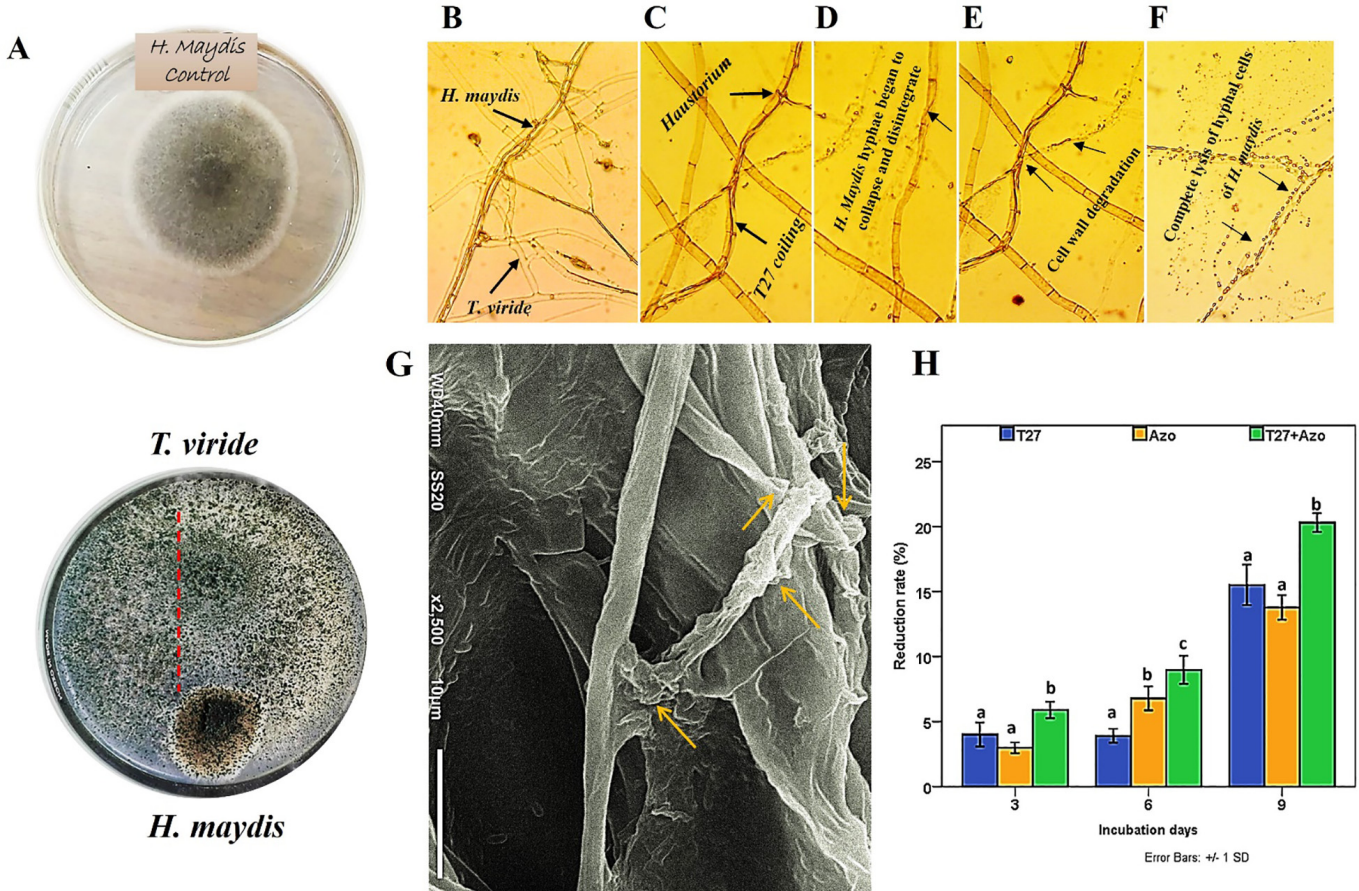
Dual culture assay and light microscopy show the interactions between the mycoparasite *T. viride* (T27) and the fungal plant pathogen *H. maydis.* **(A)** The left panel shows the growth of the pathogen and antagonist co-cultured in Petri dishes on a PDA medium. **(B, C)** The middle panel shows microscopic observations of the contact zones of the pathogen and antagonist through an initial interaction of both fungal hyphae between *T. viride* (T27) and *H. maydis*, where the former penetrates mycelial cells of the latter by haustoria and coiling. **(D-F)** The black arrow points to the degradation of hyphal cell walls of *H. maydis* due to the secretion of degrading enzymes by *T. viride* (T27) following the complete lysis of hyphal cells of *H. maydis* due to mycoparasitic attack; Magnification (800×). **(G)** Scanning electron microscopy (SEM) shows the hyphal interactions between *T. viride* and *H. maydis via* penetrating and coiling structures of *T*. *viride* hyphae around *H. maydis* hyphae (yellow arrows). The SEM picture was taken in the region where both fungi have contact, Scale bar-10 µm. **(H)** The effect of culture filtrates of *T. viride* (Blue) and *A. brasilense* (Orange), either separately or in combination (Green), on the growth rate of *H. maydis* at (3,6 and 9) incubation days. The letters above the histograms represent unique statistical groups based on ordinary one-way ANOVA (P value<0.05).

**Figure 3:**
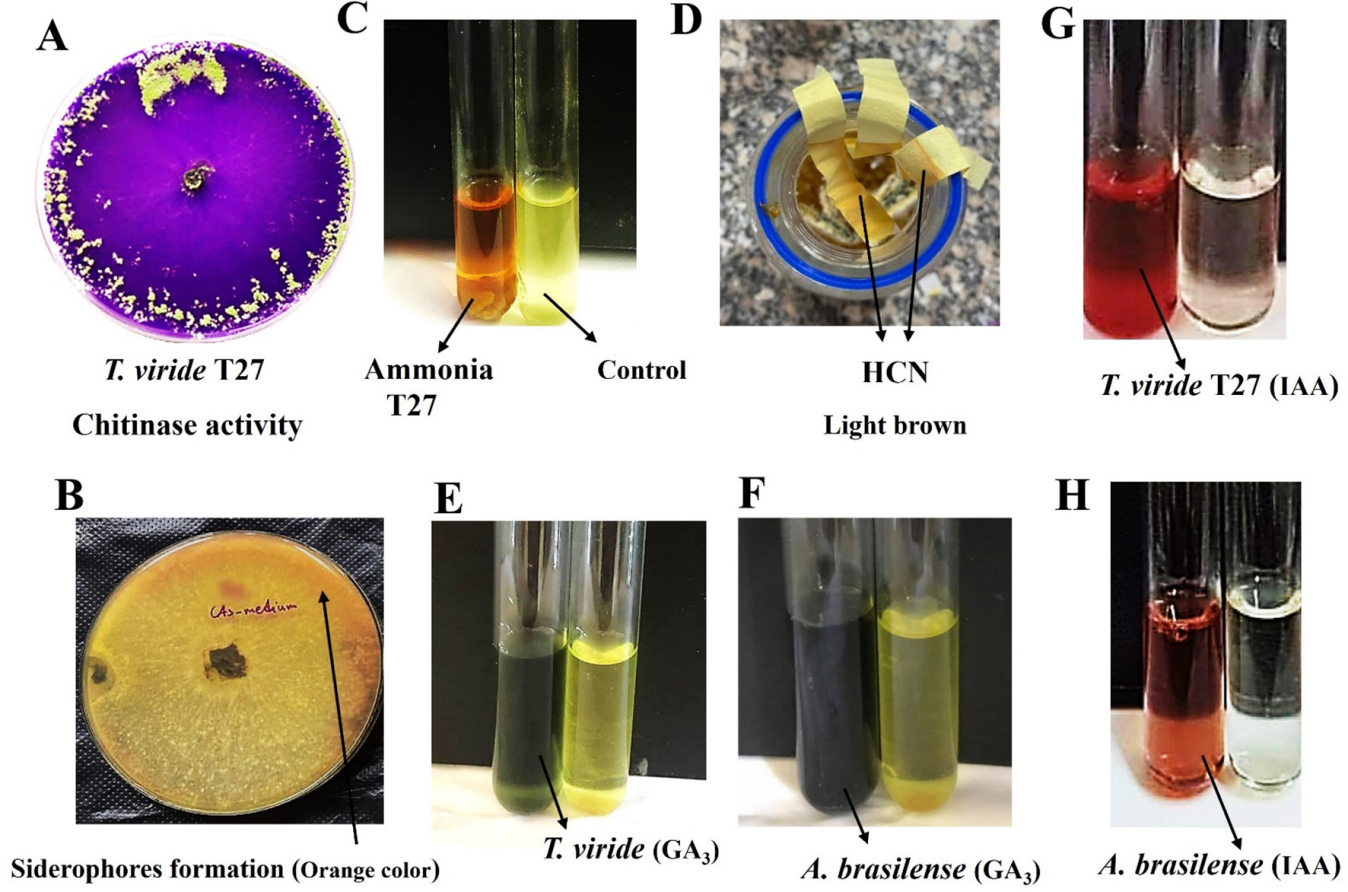
**(A)** Positive chitinase activity of *T. viride* (T27) on Lukewarm agar medium and bromocresol purple at 28°C ± 2°C for seven days. **(B)** Siderophores formation in *T. viride* was observed as a yellow-orange halo around the fungal inoculum using CAS medium (Chrome Azurol Sulfonate). **(C)** Yellow color (left) due to ammonia production by *T. viride* (T27) compared with the control (right). **(D)** A light brown color on filter paper strips indicates Hydrogen Cyanide (HCN) formation by *T. viride* (T27) using King’s B broth medium amended with glycine to detect HCN. **(E, F)** Green color (left tube) indicative of GA_3_ formation by *T. viride* (T27; E panel) and *A brasilense* (F panel). **(G, H)** IAA production (Salkowski’s reagent) of *T. viride* (T27; G panel) and *A brasilense* (H panel), both are left tubes.

The fungus, *Trichoderma viride* is one such biocontrol agent, mainly used for the control of various fungal pathogens. *T. viride* appears to be a bit granular on PDA, with green conidia distributed throughout. The cultures are typically fast growing at 28–30°C on PDA media (**Figures 1A, B**). A molecular phylogenetic tree based on rDNA internal transcribed spacers (ITS) identifies *T. viride* T27 (MH908510) by the maximum Likelihood Model of MEGA11.0 (**Figure 1C**). *T. viride* T27 (MH908510) was tested using a dual culture assay for its antagonistic activity against *H. maydis in vitro* (**Figure 2A**). The results of this assay indicated that *T. viride* grew faster than *H. maydis* and occupied the whole plate. (**Figure 2A**) showed the overgrowth of T27 upon the mycelia of *H. maydis*, where the inhibition percentage (IP) was 68.33%. Using microscopy, we also assessed the interaction area between two fungi for mycoparasitism activity. Results showed that *T. viride* approached *H. maydis*, began attaching to it, and ultimately penetrated its cell wall (**Figure 2B**). Subsequently, *T. viride* formed structures such as haustoria and appressorium that absorb nutrients from cells of the pathogenic fungus, which finally led to cell denaturation and lysis. Specifically, cell wall degrading enzymes accumulate inside the pathogenic fungal cell and cause progressive degradation of its cell wall until the fungal cell walls are entirely decomposed (**Figures 2C-G**). This may be due to the action of lytic enzymes such as chitinase and other secreted secondary metabolites by T27(**Figure 3A**).

#### 3.1.2 Cell-free filtrate assay

The inhibitory nature of secreted *T. viride* metabolites was tested, and cell-free filtrate of 7-day-old *T. viride* was examined to verify its activity against the innate growth of the phytopathogenic fungus, *H. maydis*, during three incubation periods (3, 6, and 9 days). Furthermore, the combination of *T. viride* and *A. brasilense* cell filtrates on *H. maydis* growth was also compared. Here, we found that the cell-free filtrate of *T. viride* T27 was more effective than *A. brasilense* in suppressing *H. maydis* growth during all incubation periods, with the exception of day 6. The reduction rate gradually increased from three days until it reached its maximum after nine days of incubation. In addition, the combination of the cell-free filtrates of *A. brasilense* and *T. viride* significantly suppressed the growth of *H. maydis* compared to the separate mono-treatments (**Figure 2H**).

#### 3.1.3 Bioactive secondary metabolite production

The production of secondary antifungal metabolites was evaluated in a single culture of *T. viride* and its co-culture with *H. maydis* using GC/MS. The similarity of the metabolic profiles in both single and mixed cultures is shown in (**Table 1**). Results indicated that 17 bioactive compounds were detected in the single culture of T27. Conversely, the interaction of *H. maydis* and *T. viride* stimulates the production of 10 new bioactive compounds compared to the individual culture of T27. These include cis-1,4-Cyclohexanediamine, N-methyl; 10-Undecen-1-al,2-methyl-; 7-Hexadecenal, (Z)-; 9,12-Octadecadienoyl chloride, (Z, Z)-; 2,5-Octadecadiynoic acid, methyl ester; Cyclobarbital (1); Cyclobarbital (5); Cyclobarbital (6), and Cyclobarbital (7). We also found that five compounds were inhibited in the mixed culture compared to the single culture, namely 1,2-15,16-Diepoxyhexadecane; Cyclobarbital (2); Cyclobarbital (3); Cyclobarbital (4), and Cyclobarbital (8). Otherwise, 12 compounds were detected in various proportions in single and mixed cultures. The analyzed metabolites included organic acids, aromatics, fatty acids, alcohols, esters, and hydrocarbons.

**Table 1:** GC-mass analysis of the secreted secondary metabolites by an individual culture of *T. viride* (T27) and mixed culture of *T. viride* (T27) and *H. maydis*.

Peak	RT	Name	Formula	Activity	Reference	Abundance (%)	
						***T. viride***	***T. viride* + *H. maydis***
1	8.6790	Diacetone alcohol	C_6_H_12_O_2_	Antifungal, Antibacterial, antioxidant, cytotoxic	Seddek et al., 2019	9.86	17.44
2	24.592	2,4-Di-tert-butylphenol	C_14_H_22_O	Antifungal, cytotoxic	Varsha et al., 2015	1.18	0.950
3	26.772	cis-1,4-Cyclohexanediamine, N-methyl	C_7_H_16_N_2_	Antimicrobial	Khan et al., 2020 , Adedoyin et al., 2013	ND	0.430
4	28.146	10-Undecen-1-al, 2-methyl-	C_12_H_22_O	Antimicrobial		ND	0.520
5	29.662	1,2-15,16-Diepoxyhexadecane	C_16_H_30_O_2_	Antitumor		3.05	0.420
6	32.105	Z,Z,Z-1,4,6,9-Nonadecatetraene	C_19_H_32_	No activity found		ND	0.470
7	32.294	7-Hexadecenal, (Z)-	C_16_H_30_O	Antiviral, anticancer	Khan et al., 2020	ND	0.490
8	33.095	1,2-15,16-Diepoxyhexadecane	C_16_H_30_O_2_	Antimicrobial		1.47	ND
9	33.267	9,12-Octadecadienoyl chloride, (Z, Z)-	C_18_H_31_ClO	Antifungal, Antibacterial, antioxidant	Khan et al., 2020	ND	0.320
10	33.765	2,5-Octadecadiynoic acid, methyl ester	C_19_H_30_O_2_	Antifungal, Antibacterial, cytotoxic		ND	0.790
11	33.874	β-Cholestanol	C_28_H_48_O	antioxidant	Doukyu and Ishikawa, 2020	4.04	6.020
12	34.245	Retinoic acid	C_20_H_28_O_2_	Antibacterial	Jacobo-Delgado et al., 2021; Jacobo-Delgado et al., 2023	16.0	14.28
13	34.343	1-Heptatriacotanol	C_37_H_76_O	Antifungal, Antioxidant	Kadhim et al., 2016	5.14	2.580
14	34.617	7-Methyl-Z- tetradecen -1-ol acetate	C_17_H_32_O_2_	No activity found	–	1.46	1.300
15	35.338	cis-9-Tetradecenoic acid, isobutyl ester	C_18_H_34_O_2_	antioxidant	Chirumamilla et al., 2022; Chirumamilla and Taduri, 2023	1.49	2.310
16	36.036	Z,E-2,13-Octadecadien-1-ol	C_18_H_34_O	Antifungal, antibacterial	El-Shahir et al., 2022	1.00	0.960
17	36.305	6-epi-Shyobunol	C_15_H_26_O	Antifungal, antibacterial		8.84	6.860
18	36.420	(2R,3R,4aR,5S,8aS)-2-Hydroxy-4a,5-dimethyl-3-(prop-1-en-2-yl) octahydronaphthalen-1(2H)-one	C_15_H_24_O_2_	Antifungal	Kadhim et al., 2016	0.64	0.480
19	36.712	i-Propyl 9-tetradecenoate	C_17H32_O_2_	Antifungal	Kadhim et al., 2016	0.61	0.490
20	37.364	Cyclobarbital (1)	C_12_H_16_N_2_O_3_	Antimicrobial	Basha and Goudgaon, 2021	ND	0.380
21	37.679	Cyclobarbital (2)				1.22	ND
22	39.006	Cyclobarbital (3)				2.20	ND
23	39.338	Cyclobarbital (4)				2.08	ND
24	39.590	Cyclobarbital (5)				ND	28.86
25	40.019	Cyclobarbital (6)				ND	9.180
26	42.622	Cyclobarbital (7)				ND	4.490
27	42.725	Cyclobarbital (8)				39.72	ND

ND, components Not Detected by GC.

#### 3.1.4 Production of bioactive compounds and siderophores

Various mechanisms were studied to understand the biological antifungal activity of *T. viride* strain T27 against *H. maydis* by producing certain volatile compounds such as hydrogen cyanide, ammonia, and siderophores. A color change to orange at the bottom of the T27 culture plates on CAS agar medium was considered an indicator of siderophores formation, where the concentration was 0.765% (**Supplementary Table S1**, **Figure 3B**). Moreover, Ammonia was detected using a colorimetric assay (i.e., color change to yellow) and was quantified at 3.12 mg/L (**Supplementary Table S1**, **Figure 3C**). Further, a color change to light brown in filter paper strips indicated moderate (++) HCN production, which was then quantified by measuring the OD (0.208; **Supplementary Table S1**, **Figure 3D**).

#### 3.1.5 Lytic enzymes and growth hormones

Besides the production of the bioactive compounds, three cell wall degrading enzymes (cellulase, chitinase, and amylase) were considered in this study. Qualitative analysis of chitinase activity by *T. viride* strain T27 was observed by conversion of Lukewarm agar medium to a violet color (**Figure 3A**). We observed superior production of chitinase rather than cellulase and amylase in T27 (2.206, 0.121, and 0.493 mg/mL, respectively; **Supplementary Table S1**). Conversely, the plant growth promotion activity of T27 was compared to that of *A. brasilense* through their ability to produce the plant growth hormones IAA and GA_3_. Here, *A. brasilense* produced considerable amounts of GA_3_ compared to T27, where concentrations were 46.9 and 30.8 mg/L, respectively (**Figures 3E, F**; **Supplementary Table S1**). In contrast, we found that T27 produced IAA in higher quantities than *A. brasilense*, where concentrations were 33.2 and 15.3 mg/L, respectively (**Figures 3G, H**; **Supplementary Table S1**). Taken together, we confirmed that *T. viride* produces all estimated antifungal compounds besides two growth promotors.

### 3.2 Field experiments

#### 3.2.1 Disease severity and incidence

Both microbial application and LWD progression were evaluated *via* a field trial, which designed to assess the efficacy of the biocontrol agent, *T. viride*, and the bacterial strain, *A. brasilense*, in controlling the maize LWD. Generally, no disease symptoms were recorded in uninfected plants (T1, T3, T4).

**Figure 4:**
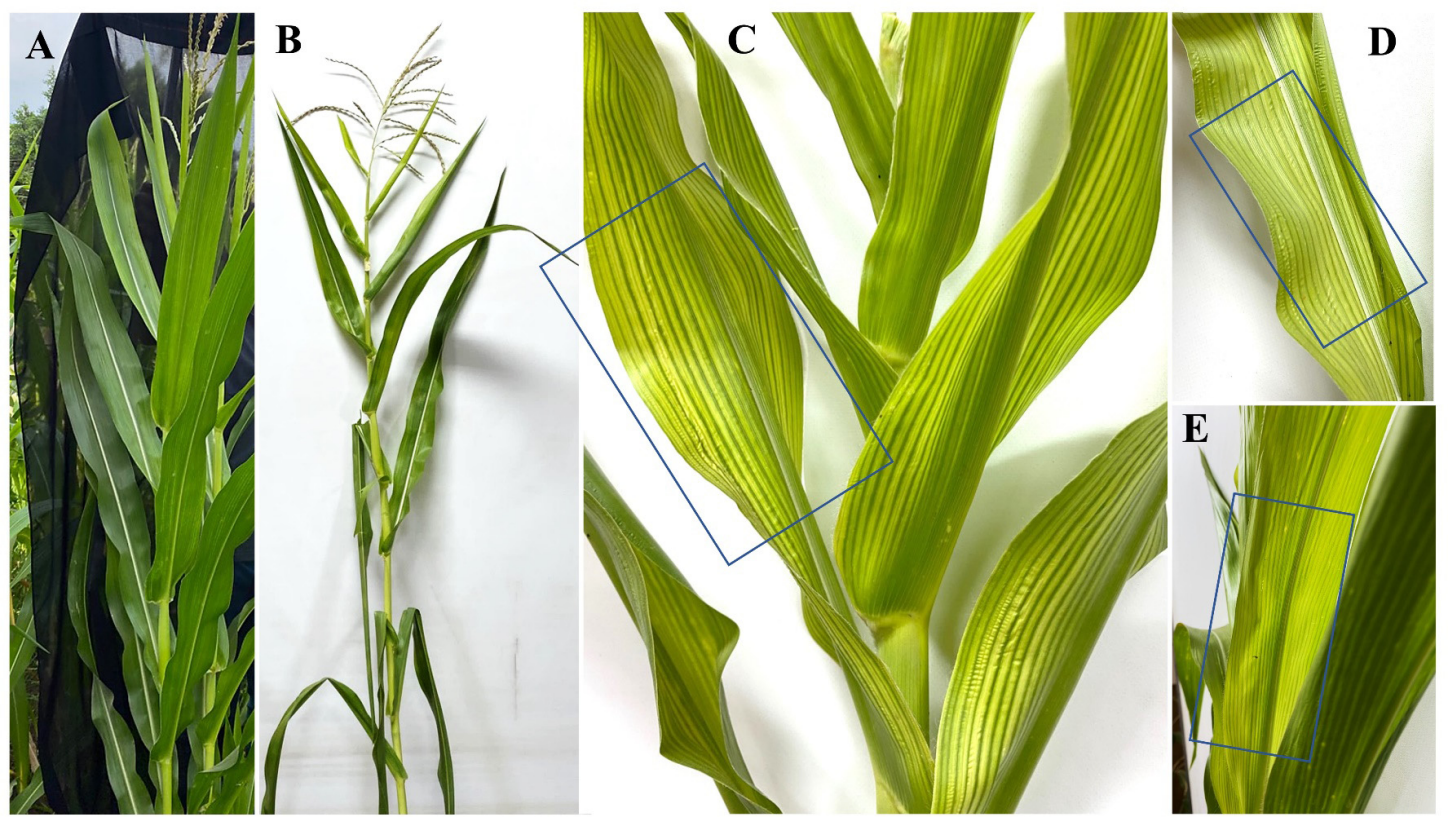
**(A)** Appearance of healthy (+ve) control maize plant and **(B)** LWD symptoms on the unhealthy (-ve) control maize plant. **(C-E)** Yellow streaks on diseased maize plant leaves.

However, the infection of maize plants with *H. maydis* resulted in disease symptoms at varying severities with or without any treatment. In contrast, uninfected control plants (+ve; T1) appeared healthy without any disease symptoms (**Figure 4A**), compared with the infected control plants (–ve; T2), which showed rapid wilting of the near ground leaves and gradually lost their color and appearance of yellow streaks on the leaves (**Figure 4B**) and the lower internodes (**Figures 4C–E**).

**Figure 5:**
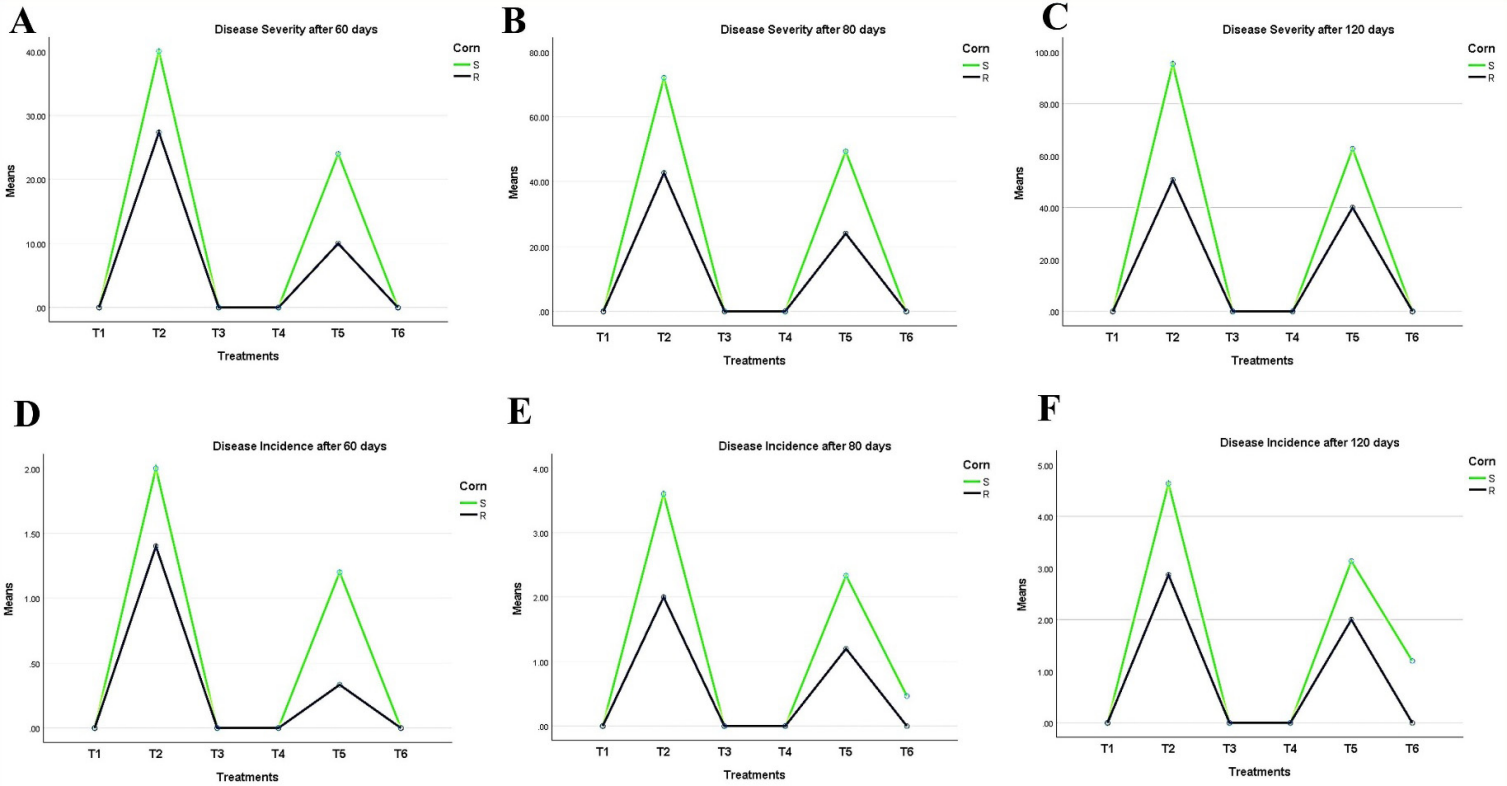
Periodic observation of LWD progression on maize hybrids after 60, 80, and 120 days of sowing shows **(A–C)** disease severity and **(D–F)** incidence. T1: Plant control (no microbes), T2: Fungal plant pathogen *H. maydis*, T3: *T. viride*, T4: *T. viride* + *A. brasilense*, T5: *T. viride + H. maydis*, T6: *T. viride* + *A. brasilense* + *H. maydis*.

Characteristically, the disease symptoms first appeared in the lower part of the plant and then spread to the upper parts over time. Data in (**Figures 5A–C**) shows two types of disease progression in the form of disease severity (DS) and disease incidence (DI) in the treated hybrid maize plants. Plants treated with *Trichoderma* T27 and *A. brasilense* separately in T3 and T4 treatments appeared healthy and vigorous (**Figures 5A–C**). Additionally, promising results were observed in the T6 treatment, which showed a high suppressing effect on DS and DI, indicating the synergistic activity between *Trichoderma* T27 and *A. brasilense* under pathogen stress. Meanwhile, treatment of infected plants with only T27 in T4 treatment showed moderate suppression of DS and DI. DS and DI data were higher in the susceptible hybrid than in the resistant plants under the same treatments. The DS and DI increased throughout the tested time intervals till they reached their maximum after 120 days of infection in the susceptible hybrid (**Figures 5A–F**).

**Figure 6:**
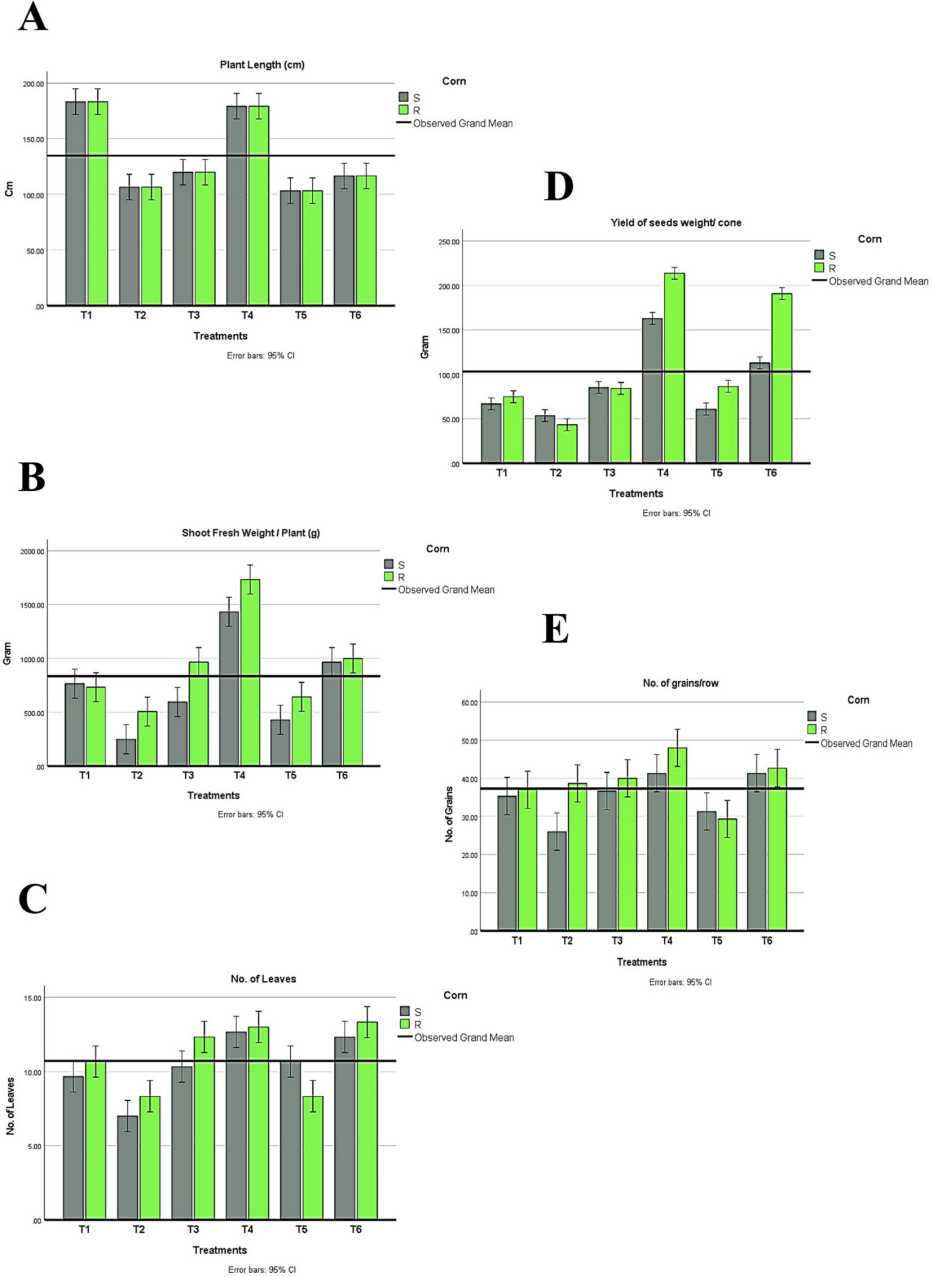
Changes in plant development under different treatments (T1-T6) of both maize hybrids showing **(A)** Plant length, **(B)** Shoot fresh weight, **(C)** Number of leaves, and **(D)** Yield of seed weight/cone and **(E)** Number of grains/rows. S: susceptible. R: resistant. T1: Plant control (no microbes), T2: Fungal plant pathogen *H. maydis*, T3: *T. viride*, T4: *T. viride* + *A. brasilense*, T5: *T. viride* + *H. maydis*, T6: *T. viride* + *A. brasilense* + *H. maydis*. The letters above the histograms represent unique statistical groups based on ordinary one-way ANOVA (P value<0.05).

**Figure 7:**
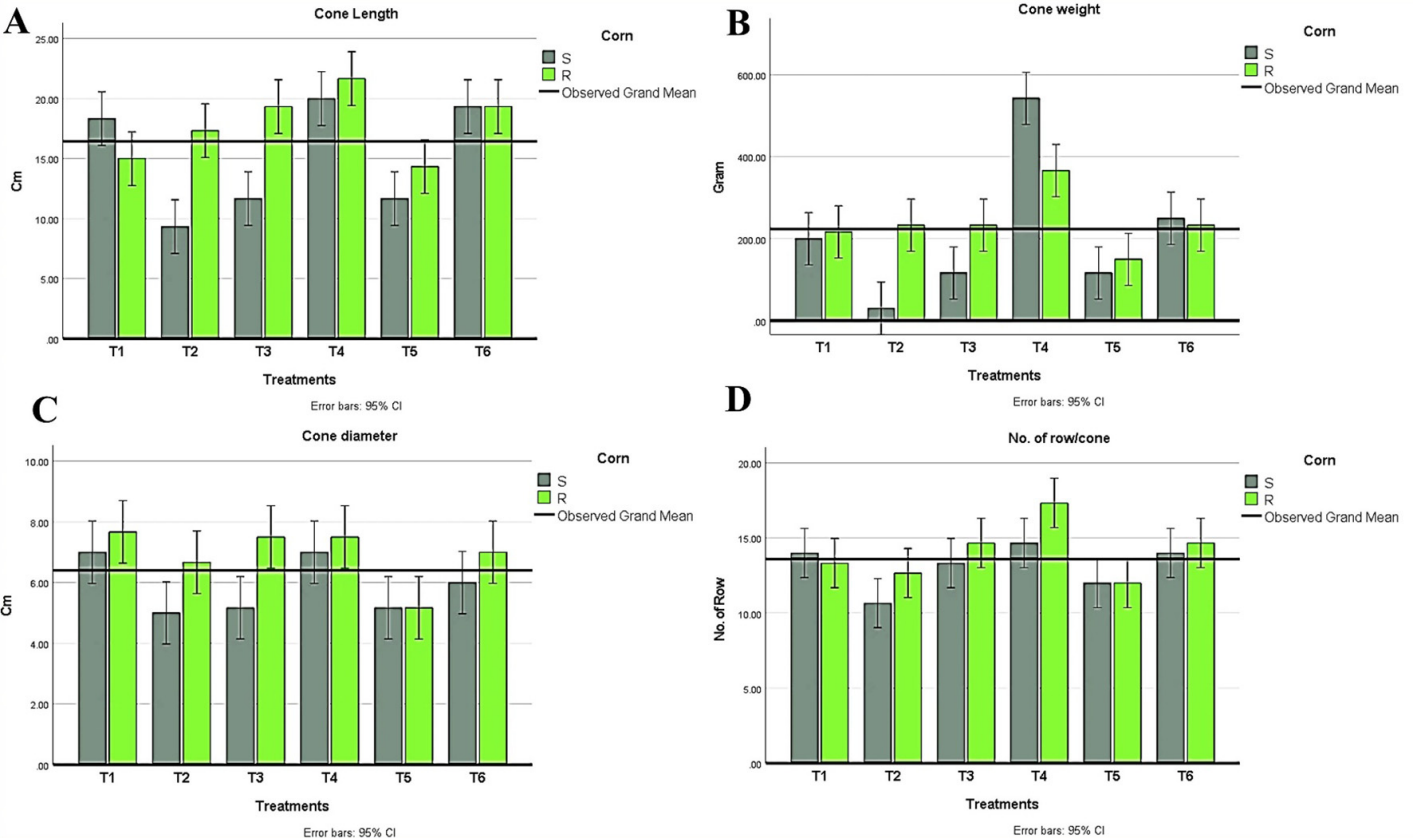
Measurements of cone parameters in all treatment conditions in both maize hybrids showing **(A)** Cone length, **(B)** Cone weight, **(C)** Cone diameter, **(D)** Number of rows/cones. T1: Plant control (no microbes), T2: Fungal plant pathogen *H maydis*, T3: *T. viride*, T4: *T. viride* + *A brasilense*, T5: *T. viride + H maydis*, T6: *T. viride* + *A brasilense* + *H maydis*. The letters above the histograms represent unique statistical groups based on ordinary one-way ANOVA (P value<0.05).

In conclusion, during the experimental trial, no symptoms were recorded in plants infected with *Azospirillum* and *Trichoderma*. Results in (**Figure 6**) were obtained from the two-way ANOVA analysis for all maize plants, which assessed parameters following inoculation with *H. maydis*, treatment with *T. viride* and/or *A. brasilense*, and their combined interactions. These data indicated that *H. maydis* significantly influences the measured parameters of both hybrids of maize plants (**Figure 6**). In addition, the weight of seeds per plant decreased by 42.8% and 32.5% for susceptible and resistant cultivars, respectively. Conversely, grain yield/plant in the treatments (T5 & T6) for the susceptible hybrid increased by 44.3% and 67%, respectively, compared to the control plants in the T2 treatment. In addition, the increment in grain yield/plant for the tolerant cultivar increased by 16.9% and 55.9% for the same treatments (**Figure 6D**). Moreover, this affects the cone criteria (i.e., length, weight, diameter, number of rows/cones, and number of grains/rows), of which the lowest values were recorded in the combined treatments (**Figure 6E**, **Figure 7**). Notably, vegetative and cone parameters were highest in T4 plants (i.e., plants treated with T27 and *A. brasilense* without *H. maydis*).

In conclusion, inoculated maize plants with *Trichoderma* alone reduced both DI and DS compared to plants without inoculation.

#### 3.2.2 Plant growth parameters, cone traits, and yield

Regarding the indices of plant development, plant height, the number of leaves, shoot fresh weight, and shoot dry weight were recorded. Interestingly, all treatments recorded higher values than plants infected with *H. maydis* only (**Figure 6**). This trend was confirmed in both susceptible and resistant hybrids. As a result of maize plant infestation with *H. maydis*, all cone traits (length, weight, diameter, number of rows/cones, number of grains/row, and seeds weight/cone (g)) were significantly affected and gave lower values in both susceptible and resistant hybrids compared to uninfected plants (**Figures 7A–D**). In addition, the weight of seeds per plant decreased by 42.8% and 32.5% for susceptible and resistant cultivars, respectively. On the contrary, the inoculation of the LWD infected-susceptible hybrid with *T. viride* only (T5) or with *A. brasilense* (T6) caused an increase in weight of seeds per cone compared to the LWD-infected plants (T2) by 44.3% and 67%, respectively. Moreover, the weight of seeds per cone of tolerant cultivar increased by 16.9% and 55.9% in inoculated plants with *T. viride* only (T5) or with *A. brasilense* (T6), respectively, compared to plants infected with *H. maydis* only (T2) (**Figure 6D**).

#### 3.2.3 Photosynthetic pigments

**Figure 8:**
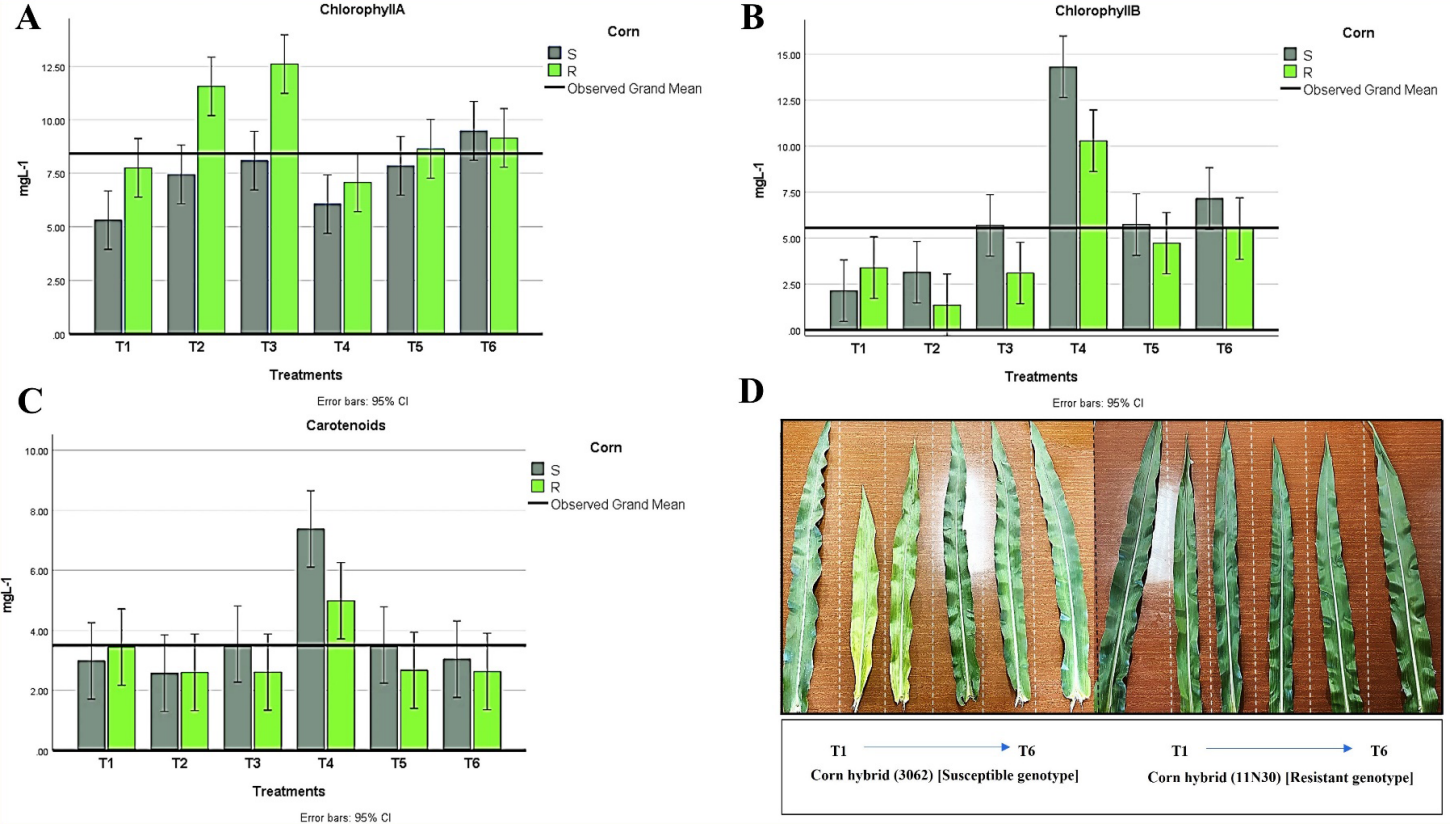
Changes in photosynthetic pigments in all treatment conditions for both maize hybrids showing **(A)** chlorophyll A, **(B)** chlorophyll B, and **(C)** Carotenoids. **(D)** Leaves have different color appearances in various susceptible and resistant hybrid treatments. T1: Plant control (no microbes), T2: Fungal plant pathogen *H maydis*, T3: *T. viride*, T4: *T. viride* + *A brasilense*, T5: *T. viride + H maydis*, T6: *T. viride* + *A brasilense* + *H maydis*. The letters above the histograms represent unique statistical groups based on ordinary one-way ANOVA (P value<0.05).

A significant difference was observed among hybrids regarding photosynthetic pigments for the foliar pigments index. The photosynthetic pigment content in maize leaves was detected in all treatments, where chlorophyll values were higher in the resistant hybrid, while chlorophyll b and carotenoid contents were higher in the susceptible hybrid (**Figures 8A–C**). Moreover, a significant decrease in chlorophyll b and carotenoids was recorded in the infected plant with *H. maydis* (T2). This trend was the opposite for chlorophyll a, which was higher in infected fungal plants compared to the control. The chlorophyll content was recorded at higher values in infected control plants than in uninfected control plants. However, we observed a significant increase in chlorophyll b and carotenoid content in T4 plants for both hybrids. These data suggest that the inoculation of maize with a mixture of *A. brasilense* and *T. viride* in the absence of *H. maydis* caused a significant increase in chlorophyll b and carotenoid content compared to control plants in both hybrids (**Figure 8D**). Additionally, results showed that carotenoids increased in maize leaves inoculated with *Trichoderma* and *Azospirillum* in the presence or absence of *H. maydis* but decreased in maize plants infected with *H. maydis* only (**Figure 8C**).

#### 3.2.4 Oxidative enzymes

Regarding oxidative enzymes, we showed that chitinase and CAT activities were significantly higher in control plants infected with *H. maydis* (**Supplementary Table S2**). However, no significant differences were observed in PO activity among various treatments. PPO activity was higher in T4 and T6 plants than in T5 plants. These results showed the impact of *Azospirillum* treatment on pathogen suppression compared with the *Trichoderma* alone and control with or without the pathogen. Furthermore, PPO and POD activity was higher in resistant hybrids than in susceptible plants.

#### 3.2.5 Maize cross-section root analysis

**Figure 9:**
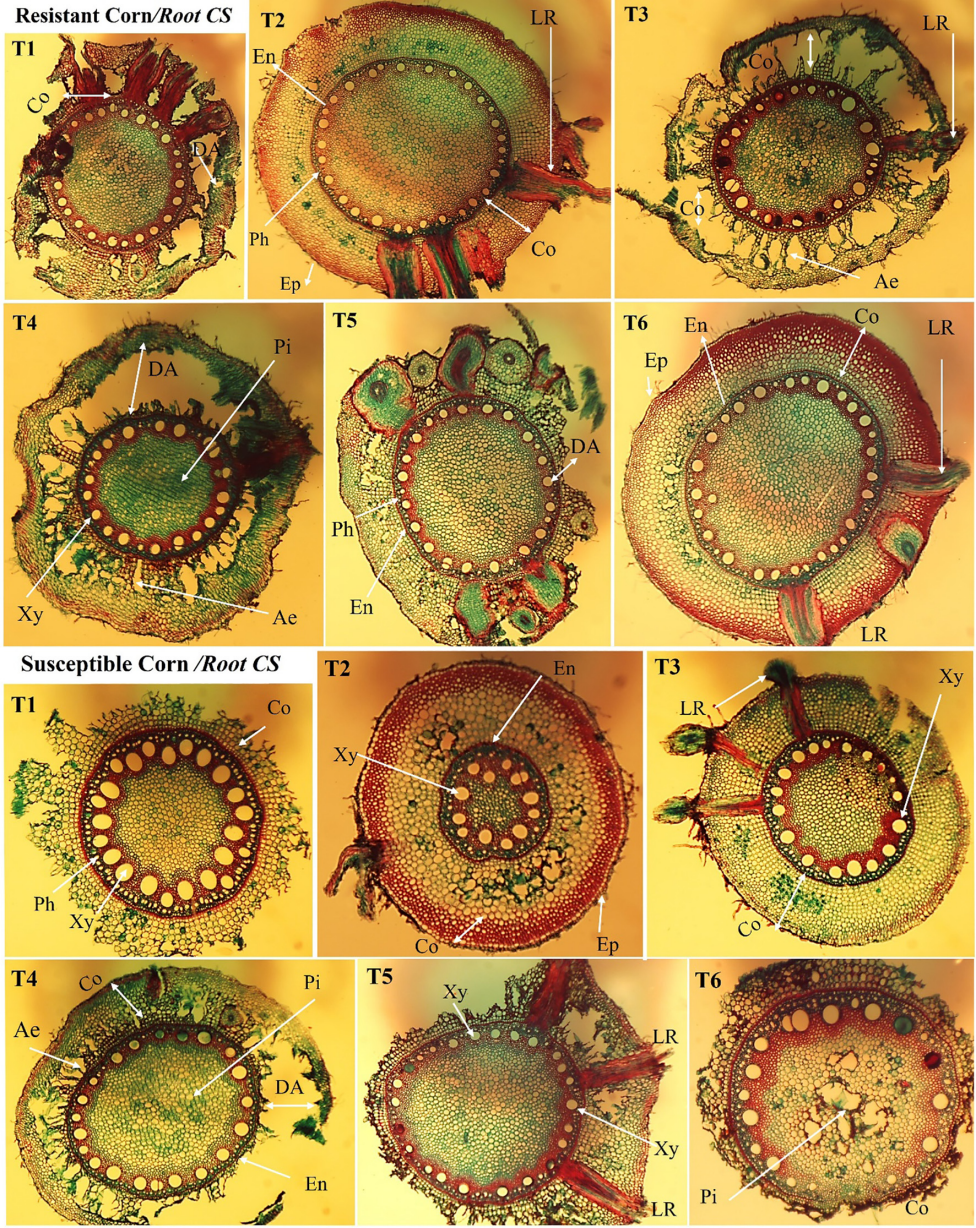
Anatomical study of the cross-section of cultivated maize root under different treatments (T1–T6). The root cross-section of an inoculated plant between two maize hybrids shows different root measurements in the maize epidermis, endodermis, phloem, and xylem for the resistant and susceptible hybrids. *LR*: lateral roots, *Ep*: epidermis, *Co*: cortex, DA: degradation area, *En*: endodermis, *Xy*: xylem, *Ae*: aerenchyma, *Pi*: pith, and *Ph*: phloem; Magnification (100×). T1: Plant control (no microbes), T2: Fungal plant pathogen *H. maydis*, T3: *T. viride*, T4: *T. viride* + *A. brasilense*, T5: *T. viride + H. maydis*, T6: *T. viride* + *A. brasilense* + *H. maydis*.

Infection of maize roots with *H. mayids* decreased whole root thickness by 40.9% and 34.5% compared to control plants in susceptible and resistant hybrids, respectively. Notably, the combination of *T. viride* and *A. brasilense* enhanced the root thickness in the presence or absence of *H. mayidism*. We also noted that the diameter of the phloem and pith and the endodermis thickness was thinner in *H. mayids-*infected roots than in other treatments, and the same trend was observed in both hybrids. Moreover, we also found that susceptible hybrids increased the number and thickness of their xylem vessels compared to resistant hybrids. The highest degradation area of the root cortex was observed in *H. maydis-*infected plants in both hybrids. We also noted changes in the cortex area with plant response to fungal infection. Lateral root (LR) development was also observed in plants infected with *T. viride* only or *H. maydis* in a suspectable hybrid. Conversely, the recorded number and thickness of aerenchyma (i.e., air pockets forming in the cortex cell layers that may aid in gas exchange) was lower in *H. maydis-*infected plants (**Supplementary Table S3**; **Figure 9**). Taken together, we consider *T. viride* to be a potentially promising approach as a safe and effective natural fungicide compared to other chemical fungicides due to its potent suppression of LWD in maize.

## 4 Discussion

*Trichoderma viride* MH908510 (T27) had significant antagonistic activity against *H. maydis* in both solid and liquid media. *T. viride* is a fast-growing and highly sporulating strain that overgrew and covered the entire surface of the medium plate during the first three days of incubation, thereby limiting the growth of *H. maydis* (Hariharan et al., 2022; Arif et al., 2023). Furthermore, mycoparasitism is another behavior of numerous *Trichoderma* strains that enables them to attack plant pathogens (Druzhinina et al., 2011; Hewedy et al., 2020a; Woo et al., 2023; Kumari et al., 2024). We confirmed that the antifungal activity of T27 against *H. maydis* by the production of volatile compounds (NH_3_ and HCN) as well as siderophores (**Figures 3B–D**) (Saadaoui et al., 2023). Alternatively, HCN might inhibit *H. maydis* growth by inhibiting cytochrome C oxidase and blocking the respiratory electron transport chain (Walia et al., 2021; Lahlali et al., 2022; Bhadrecha et al., 2023). Several studies have reported that NH_3_ might have had a fungistatic role in inhibiting conidial germination and inducing endoplasmic reticulum stress, which might suppress protein synthesis (Avalos et al., 2020; Liu et al., 2021). Likewise, NH_3_ could induce oxidative stress and/or cell apoptosis in pathogenic fungi tissues (Missall et al., 2004; Görg et al., 2019; Liu et al., 2021). Moreover, HCN can be converted into NH_4_ by the cyanide dioxygenase system (Luque-Almagro et al., 2018), which promotes plant growth. Thus, biocontrol agents can inhibit the growth of pathogenic fungi through their ability to form biofilms and reduce pathogenic root colonization, as well as siderophore production (Howell et al., 1988; Singh et al., 2019). *A. brasilense* (diazotrophic bacteria) is widely applied in the cultivation of cereal crops, especially maize, wheat, and rice, as a nitrogen fixer, rock minerals solubilizer, and plant growth promoter, and it also has a significant role as an antimicrobial agent (Bashan and De-Bashan, 2010; Galindo et al., 2022). Moreover, T27 stimulated plant growth by producing GA_3_ and IAA, which are implicated in phytonutrient availability. Thus, the combination of T27 and *A. brasilense* supplies various pathways for phytonutrient availability and enhancement of the plant defense system toward *H. mayids* (Olanrewaju et al., 2017; Cui et al., 2024). The GC/MS data (**Table 1**) showed that the *T. viride* culture had more diacetone alcohols in the co-culture than in the *T. viride* culture alone under biotic stress. This confirms that the *T. viride* filtrate was a good suppressor for *H. mayids*. These results were aligned with previous reports indicating that ketones and alcohol are cytotoxic, in addition to their inhibitory effect on pathogenic fungi by delaying their conidial growth (Vinale et al., 2008; Seddek et al., 2019). Cis-1,4-Cyclohexanediamine, N-methyl; 10-Undecen-1-al, 2-methyl-; 1,2-15,16-Diepoxyhexadecane and 7-Hexadecenal, (Z)- were identified as significant components in the co-culture, which suggests that *T. viride* was able to produce these compounds in the presence of *H. mayids*, concluding that these compounds have antimicrobial activity (Kadhim et al., 2016; Li et al., 2022). Additionally, others have also detected isopropyl e-9-tetradecenoate among 31 bioactive compounds found in *Candid albicans* culture (Brighenti et al., 2017), which we also detected in both cultures in this work. Furthermore, 6-epi-shyobunol and cis-13-Octadecenoic acid have previously been reported as antifungal compounds (El-Shahir et al., 2022). LWD is typified by the rapid wilting of maize leaves, which firstly become faint green before they entirely lose color and dry with inward rolling from the edges. Lastly, the whole plant becomes dry with yellow-brown discoloration of the vascular bundles, followed by the appearance of red-brown stripes advancing up to the fifth internode or further up (Degani and Cernica, 2014; Ortiz-Bustos et al., 2016; Degani and Dor, 2021). Therefore, a field experiment was designed to develop an alternative biological control strategy for LWD in maize. *T. viride* was used as a biocontrol agent, and *A. brasilense* was used as a plant growth promoter to minimize both disease severity and incidence. In addition, a highly significant suppression was observed when maize plants were inoculated with *T. viride* combined with *A. brasilense*. Similarly, previous studies have also reported the potential of this combination as a biocontrol agent (Karthika and Vanangamudi, 2013; Khalil and Shimaa, 2020). These microbes protect maize plants against LWD *via* hydrolytic enzymes that inhibit the growth of the pathogenic fungi, as well as their ability to colonize plant roots (Degani, 2021; Degani and Dor, 2021). By tracking the effects of *H. maydis* on physiological changes in maize plants, we found that the production of photosynthetic pigments was a remarkable indicator of the plant’s response to biotic stress (Marín-Ortiz et al., 2020; Jha and Mohamed, 2023). Due to this fungal infection, the noticeable decrease in photosynthetic pigments in *H. maydis*-infected maize disrupts the enzymes responsible for pigment production, thus reducing their production rate and increasing their degradation rate (Horst et al., 2008; Matei et al., 2018). In both maize hybrids, the formed cone parameters (length, weight, diameter, the number of rows/cones, the number of grains/row) were significantly decreased as a result of plant infection with *H. maydis*, and these findings were consistent with published work (Degani and Cernica, 2014; Ortiz-Bustos et al., 2016). In addition, we also found that the fungal infection negatively affected the photosynthetic pigments as well as caused a decrease in the efficiency of the photosynthesis process due to the change in leaf anatomy under stress conditions such as leaf pruning, chlorosis and reduction of leaf area (Drori et al., 2013; Sunitha et al., 2020). Indeed, auxin and cytokinin enhanced the function of the root system, which increased water and nutrient availability to other parts of the plant, especially the leaves, thus enhancing photosynthetic pigment production (Werner et al., 2010; Bielach et al., 2017). Furthermore, the combined treatment of maize plants with T27 and *A. brasilense* modified the plant cell structure and physio-biochemical reactions, resulting in the synthesis of proteins and enzymes associated with different pigment stabilities and protection of carotenoids from oxidation (Pal et al., 2021). Since the bacterium *Azospirillum* fixes atmospheric nitrogen in the form of ammonia, which is considered a precursor of glutamate formation, it is consequently used in the synthesis of chlorophyll in most plants (Costa et al., 2015; Fukami et al., 2018). Interestingly, our results showed a significant difference between maize hybrids regarding photosynthetic pigments, possibly due to genetic variability for disease susceptibility (Campos et al., 2021). Previous studies have shown the production of oxidative enzymes in stressed plants. PO and CAT were formed to remove the accumulated reactive oxygen species (ROS), such as H_2_O_2_, produced due to pathogen invasion (Feng et al., 2022) (**Supplementary Table S2**). Additionally, PO is required for synthesizing phenolic compounds, re-building the plant cell wall at infection sites, and synthesizing ethylene (Magbanua et al., 2007; Terna et al., 2022). In addition, our results were agreed by (Pereyra et al., 2010), who reported that the oxidative enzymes increased in *Azospirillum*-treated plants because this bacterium activated the plant antioxidant system and increased the activity of antioxidant enzymes, which scavenged or reduced ROS in the maize plants and provided better growth conditions. Considering our results, PAL has a regulatory role in defense mechanisms against fungal pathogen attacks because it plays an essential role in the biosynthesis of phenolics. Accordingly, the high PAL activity is associated with the accumulation of phenolic compounds in plant tissues (Aoun, 2017; Jiang et al., 2019). Furthermore, *Trichoderma* can alter several physiological operations, including transpiration, stomatal conductance, water use efficiency, nutrient uptake, and balancing the phytohormones changes, as well as improving their capacity to suppress fungal diseases (Swain and Mukherjee, 2020; Yu et al., 2021). Maize plants secret massive amounts of secondary metabolites as root exudates that protect plants from pathogens, and some act as botanical fungicides (Elshahawy and Khattab, 2022). These metabolites damage the fungal cell walls and membranes, inhibiting spore germination, mycelial development, germ tube elongation, sporulation, and synthesizing enzymes, DNA, and proteins (Yoon et al., 2013). Accordingly, the development of cone traits in both hybrids (length, weight, diameter, the number of rows/cones, the number of grains/row) was significantly decreased as a result of plant infection with *H. maydis*, and this was consistent (Sunitha et al., 2020). In contrast, the cone traits were enhanced in maize plants inoculated with *T. viride* and *A. brasilense*, compared to previous reports for the inoculation of maize plants with *Trichoderma* and *A. brasilense* (Akladious and Abbas, 2012; Galindo et al., 2022), which enhanced cereal growth parameters like root and shoot dry weight, root and shoot fresh weight, leaf number, plant height, root, and shoot length. Following the histological characterization of the maize roots, a reduction in root tissue diameters was observed in infected plants as a defense mechanism to slow the spread of pathogens to other plant parts. In contrast, the number, diameter, and thickness of xylem vessels were higher in *H. maids-*infected plants than in different treatments (**Figure 9**). Plants with wider xylem vessels were more susceptible to diseases than those with narrower xylem diameters (Pouzoulet et al., 2017). Pathogenic fungi degradation of the root cortex could result from the activity of the synthesized cell wall degrading enzymes to promote tissue invasion and colonization (Terna et al., 2022). The interaction between associated microbes with roots, whether beneficial or pathogenic, and their phenotypes is vital to their ability to avoid diseases (Lynch, 2019). *H. maydis* infected maize plants led to an increase in whole root thickness, cortex area degradation, and pith thickness for resistant maize hybrids in comparison to plants inoculated with *A. brasilense* and/or *T. viride*, which decreased cortex thickness due to their beneficial effects on inhibiting the pathogenic fungus and restoring the healing of the roots (**Figure 9**). Furthermore, the inoculation of maize plants with *T. viride* and A. *brasilense* reduced the cortex area, enhancing the root’s ability to reduce respiration and promote deeper roots, enhancing plant growth and grain yield (Chimungu et al., 2015; Chaudhary et al., 2022). Therefore, *T. viride* strain T27 is vital for genetically protecting cereal crops against several pathogenic fungi. However, further experiments are needed to determine the effectiveness of these endophytic microbes under different field conditions. Ultimately*, T. viride* and *A. brasilense* are multifunctional allies for maize growth under biotic stress.

## 5 Conclusion

Indeed, host-pathogen interaction is a highly dynamic process between phytopathogenic microbes and their host plants. Thus, in this study, we sought to improve and establish an environmentally friendly and consistent method to control the development of phytopathogenic disease. The combination of *T. viride* and *A. brasilense* in protecting maize crops infected with *H. maydis*, was studied *in vitro* and the field*. T. viride* strain T27 significantly displayed antagonistic activities against *H. maydis* using different mechanisms. Notably, lytic enzymes produced by *T. viride* T27 and the bioactive secondary metabolitesplayed a vital role in suppressing the pathogen,. Maize plants treated with T27 and *A. brasilense* alone or in combination showed remarkable potential for suppressing LWD and promoting plant growth. These findings suggest that corn seed coating with beneficial fungi and/or bacterial endophytes supports mutualistic colonization. Additionally, *de novo* transcriptome assembly, functional annotation, and expression profiling of the root system inoculated with *T. viride* are necessary.

## Data Availability

The original contributions presented in the study are included in the article/**Supplementary Material**. Further inquiries can be directed to the corresponding author.
