# Supplementary material for: Co-inoculation of Trichoderma viride with Azospirillum brasilense could suppress the development of Harpophora maydis-infected maize in Egypt
Source: Front Plant Sci. 2025 Feb 6;15:1486607. doi: 10.3389/fpls.2024.1486607 (PMC11839624; doi:10.3389/fpls.2024.1486607)
Supplement: Supplementary file 1 [file DataSheet1.docx]

**Supplementary data**

**
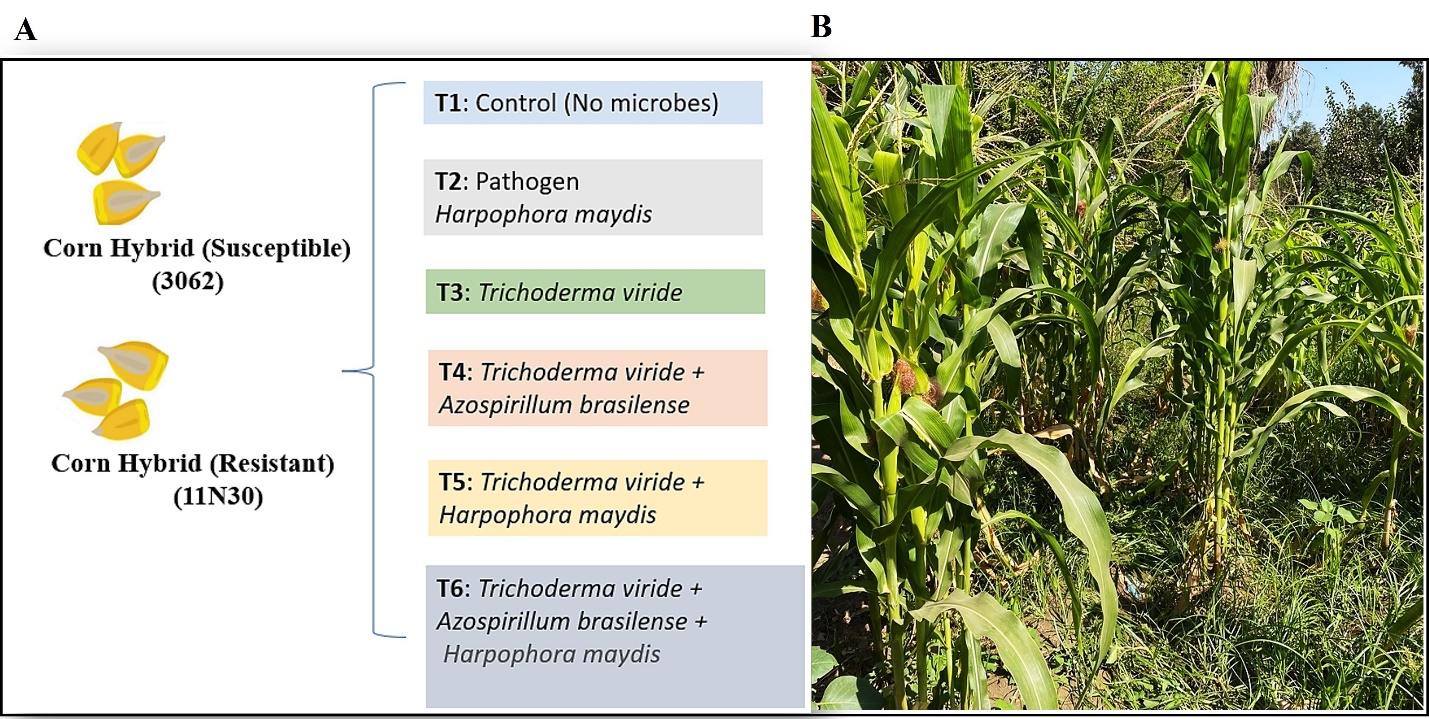
**

**Figure S1: (A)** Experimental field design showing various treatments with two maize hybrids. **(B)** Maize plants with cobs.

**Table S1**. Quantities of volatile compounds, siderophores, lytic enzymes, and growth hormones of *T. viride* (T27) and *A. brasielence*.

| **Microbes** | **Volatile compounds** | |  | **Lytic enzymes (mg/mL)** | | | **Growth hormones**  **(mg/L)** | |
| --- | --- | --- | --- | --- | --- | --- | --- | --- |
|  | HCN  (OD) | NH_4_  (mg/L) | Siderophores  (%) | Chitinase | Cellulase | Amylase | IAA | GA_3_ |
| ***T. viride* (T27)** | 0.208 | 3.12 | 0.765 | 2.206 | 0.121 | 0.493 | 33.2 | 30.8 |
| ***A. brasilense*** | - | - | - | - | - | - | 15.3 | 46.9 |

**Table S2**. The oxidative enzymes of two maize hybrids under different treatments.

| **Treatments** | | **T1** | **T2** | **T3** | **T4** | **T5** | **T6** | **df** | **F** | **P** |
| --- | --- | --- | --- | --- | --- | --- | --- | --- | --- | --- |
| **Oxidative enzymes** | | | | | | | |  |  |  |
| **Polyphenol** | Treatments | 0.93**^ab^** | 0.79**^c^** | 1.06**^ab^** | 1.24**^ab^** | 0.89**^c^** | 1.35**^a^** |  |  |  |
|  |  |  |  |  |  |  |  | 5 | 4.28 | .006 |
|  | Corn | 1 | | | | | | | 8.95 | .006 |
|  | Treatments × Corn | 5 | | | | | | | 3.43 | .017 |
| **Peroxidase** | Treatments | 173.00**^a^** | 179.00**^a^** | 178.80**^a^** | 172.36**^a^** | 183.00**^a^** | 178.58**^a^** | 5 | 1.89 | .13 |
|  | Corn | 1 | | | | | | | 13.65 | .001 |
|  | Treatments × Corn | 5 | | | | | | | 4.05 | .008 |
| **Chitinase** | Treatments | 30.47**^b^** | 48.18**^a^** | 39.59**^ab^** | 36.02**^b^** | 47.25**^a^** | 29.64**^b^** | 5 | 9.74 | .670 |
|  | Corn | 1 | | | | | | | 4.86 | .169 |
|  | Treatments × Corn | 5 | | | | | | | 19.21 | .800 |
| **Phenyl** | Treatments | 94.78**^b^** | 78.51**^cd^** | 107.15**^a^** | 72.06**^d^** | 85.70**^bc^** | 86.11**^bc^** | 5 | 29.86 | <.001 |
|  | Corn | 1 | | | | | | | 36.52 | <.001 |
|  | Treatments × Corn | 5 | | | | | | | 14.93 | <.001 |
| **Catalase** | Treatments | 12.14**^ab^** | 13.93**^a^** | 12.56**^ab^** | 10.14**^c^** | 13.25**^ab^** | 11.45**^ab^** | 5 | 2.98 | .031 |
|  | Corn | 1 | | | | | | | 0.425 | .521 |
|  | Treatments × Corn | 5 | | | | | | | 4.046 | .008 |

According to Duncan's multiple range test, values within a raw followed by the same letter (s) are not significantly different at the P = 0.05 level.

**Table S3**. Anatomical measurements in roots of two maize hybrids under different treatments.

| **Hybrids** | **Treatments** | The whole thickness of the root (µ) | **Cortex** | | Endodermis thickness (µ) | Pith thickness (µ) | **Xylem** | | Phloem  Thickness (µ) | **Aerenchyma** | |
| --- | --- | --- | --- | --- | --- | --- | --- | --- | --- | --- | --- |
|  |  |  | Thickness (µ) | Degradation area thickness (µ) |  |  | Thickness (µ) | Number of Vessels in rows |  | Thickness (µ) | Number |
| **Susceptible** | T1 | 1980 | 450 | -- | 45.0 | 720 | 18.0 | 2 | 22.0 | 55 | 24 |
|  | T2 | 1170 | 315 | 360 | 18.0 | 270 | 45.0 | 3 | 18.0 | 42 | 14 |
|  | T3 | 1198 | 360 | -- | 27.0 | 630 | 27.0 | 2 | 27.0 | 60 | 18 |
|  | T4 | 2430 | 450 | -- | 28.0 | 1080 | 36.0 | 2 | 45.0 | 62 | 22 |
|  | T5 | 1458 | 405 | 225 | 22.5 | 1107 | 9.00 | 2 | 31.5 | 72 | 24 |
|  | T6 | 2178 | 203 | 225 | 27.0 | 900 | 18.0 | 2 | 40.5 | 81 | 20 |
| **Resistant** | T1 | 2925 | 540 | -- | 24.0 | 1008 | 39.5 | 2 | 31.5 | 90 | 26 |
|  | T2 | 1917 | 513 | 2881 | 18.1 | 1395 | 16.3 | 2 | 27.0 | 83 | 25 |
|  | T3 | 2430 | 585 | 90.2 | 27.2 | 900 | 18.0 | 3 | 30.1 | 108 | 29 |
|  | T4 | 3240 | 540 | -- | 27.0 | 765 | 22.1 | 3 | 27.0 | 90 | 26 |
|  | T5 | 2163 | 378 | 360 | 27.1 | 821 | 23.2 | 3 | 38.7 | 90 | 25 |
|  | T6 | 2718 | 450 | 252 | 28.8 | 1188 | 24.1 | 3 | 32.1 | 99 | 30 |
